# The Endosomal Sorting Complex, ESCRT, has diverse roles in blood progenitor maintenance, lineage choice and immune response

**DOI:** 10.1242/bio.060412

**Published:** 2024-06-18

**Authors:** Arindam Ray, Yashashwinee Rai, Maneesha S. Inamdar

**Affiliations:** ^1^ Molecular Biology and Genetics Unit, Jawaharlal Nehru Centre for Advanced Scientific Research, Jakkur, Bangalore 560064, India; ^2^ Institute for Stem Cell Science and Regenerative Medicine (DBT-inStem), GKVK Post, Bellary Road, Bangalore 560065, India

**Keywords:** Hematopoiesis, ESCRT, Lineage choice, Notch signaling, Ubiquitin accumulation, Lamellocytes, Cargo sorting, Non-canonical function of ESCRT

## Abstract

Most hematological malignancies are associated with reduced expression of one or more components of the Endosomal Sorting Complex Required for Transport (ESCRT). However, the roles of ESCRT in stem cell and progenitor maintenance are not resolved. Parsing signaling pathways in relation to the canonical role of ESCRT poses a challenge. The *Drosophila* hematopoietic organ, the larval lymph gland, provides a path to dissect the roles of cellular trafficking pathways such as ESCRT in blood development and maintenance. *Drosophila* has 13 core ESCRT components. Knockdown of individual ESCRTs showed that only Vps28 and Vp36 were required in all lymph gland progenitors. Using the well-conserved ESCRT-II complex as an example of the range of phenotypes seen upon ESCRT depletion, we show that ESCRTs have cell-autonomous as well as non-autonomous roles in progenitor maintenance and differentiation. ESCRT depletion also sensitized posterior lobe progenitors to respond to immunogenic wasp infestation. We also identify key heterotypic roles for ESCRT in position-dependent control of Notch activation to suppress crystal cell differentiation. Our study shows that the cargo sorting machinery determines the identity of progenitors and their adaptability to the dynamic microenvironment. These mechanisms for control of cell fate may tailor developmental diversity in multiple contexts.

## Teaser

**Summary:** Components of the cargo-sorting machinery have context-dependent roles in blood progenitor maintenance and lineage choice, helping to tackle immune challenges and tailor developmental diversity.

## INTRODUCTION

Tissue patterning requires spatiotemporally controlled cell proliferation, progenitor specification and lineage differentiation. While a limited number of signaling circuits impact these complex cell properties, their regulation is highly context-dependent. Endocytic trafficking integrates extracellular cues with intracellular changes to maintain, attenuate or amplify signaling (Di Fiore and von Zastrow, 2014). The stereotypical roles of the endocytic machinery in transport and cargo sorting converge to generate complex signaling, thereby significantly impacting tissue homeostasis. However, due to their overlapping roles, tissue and cell-type-specific functions of the sorting machinery components have been difficult to dissect out *in vivo*.

Protein trafficking and turnover through the endo-lysosomal route allows rapid post-translational modulation of signal transduction. The conserved ESCRT machinery actively controls the sorting of ubiquitinated cargoes for lysosomal degradation. This complex consists of four hetero-oligomeric subunits (ESCRT-0, I, II and III) that sequentially bind to endomembrane-bound ubiquitinated cargoes in order to sequester them into intraluminal vesicles (ILV) of the multivesicular bodies/endosomes (MVB/MVE). The *Drosophila* ESCRT comprises 13 core components (Vaccari et al., 2009; Alfred and Vaccari, 2016). ESCRT-0 (Hrs, Stam) binds to the ubiquitinated cargoes through a ubiquitin-interacting motif. It then recruits ESCRT-I (Vps28, Tsg101, Vps37A, Vps37B) and ESCRT-II (Vps25, Vps22 and Vps36), which act as a bridging complex to assemble ESCRT-III (Vps32, Vps24, Vps20, Vps2). ESCRT-I-dependent inward membrane budding and ESCRT-III-dependent membrane scission lie at the heart of endosomal protein sorting (Radulovic et al., 2018). Vps32 is the principal filament-forming component that undergoes activation and polymerization upon binding with various nucleating factors and integrates previous steps of endosomal sorting (Vietri et al., 2019). The final step involves disassembly of ESCRT subunits and scission of the membrane neck of the intraluminal vesicles, which is mediated by the Vps4-Vta1 mechanoenzyme complex.

Though phenotypic diversity of ESCRT mutants is rare in unicellular organisms like budding yeast, dysfunction of metazoan ESCRT components can manifest as distinct and diverse cellular and histological phenotypes such as defective MVB biogenesis and incorrect cell fate choice, tissue hyperproliferation, apoptotic resistance, neoplastic transformation etc., due to dysregulated activation of signaling pathways such as Notch, EGFR and JAK/STAT, thereby altering tissue homeostasis (Vaccari et al., 2009; Herz et al., 2009; Tognon et al., 2014; Vietri et al., 2019). The range of functional outputs of ESCRT modulation makes it an interesting target to explore in the context of stem and progenitor cell lineage choice.

While several endocytic proteins such as Atg6 (Shravage et al., 2013), WASH (Xia et al., 2014), Rabex-5 (Reimels and Pfleger, 2015), Rab5, Rab 11 (Yu et al., 2021), Asrij (Kulkarni et al., 2011) and ARF-1 (Khadilkar et al., 2014) are implicated in developmental signaling and blood cell homeostasis, little is known about the role of ESCRT in hematopoiesis. Previous genetic screens and knockout-based functional analyses in both *Drosophila* and mouse models showed a role of ESCRT in maturation of specific blood cell types in erythroid and lymphoid lineages and a possible functional link of ESCRT to blood cell homeostasis (Avet-Rochex et al., 2010; Adoro et al., 2017; Liu et al., 2021). Hence, we analyzed the role of ESCRT in blood cell homeostasis, using the *Drosophila* lymph gland model.

The larval blood progenitors in *Drosophila* reside in the multi-lobed lymph gland that flanks the cardiac tube in segments T3 and A1. The primary lobe has a medullary zone enriched in progenitors, a cortical zone of differentiated blood cells (plasmatocytes, crystal cells and lamellocytes) and the hematopoietic niche (posterior signaling center). Blood progenitors of *Drosophila* are linearly arranged in primary, secondary and tertiary lobes of the lymph gland and are characterized by the expression of several markers such as Domeless, TepIV and DE-Cadherin. The lymph gland develops in an anterior to posterior sequence, with younger progenitors in the posterior lobes (Rodrigues et al., 2021a), allowing complete sampling of the progenitor pool. Previous studies showed heterogeneity of the progenitor population in gene expression, mitochondrial morphology and dynamics, signaling, differentiation potential and immune function (Rodrigues et al., 2021a; Ray et al., 2021). Posterior progenitors are refractile to immune challenge due to differential activation of JAK/STAT and Notch signaling (Rodrigues et al., 2021a; Ray et al., 2021). Thus, the lymph gland is an accessible model representing the complexities of tissue homeostasis. Hence, we investigated the role of ESCRT components in spatiotemporal control of blood progenitor homeostasis and myelopoiesis in the lymph gland.

Here, we elucidate the role of all 13 *Drosophila* core ESCRT components in ubiquitinated cargo sorting and blood cell lineage choice across progenitor subsets. We show that though ubiquitous, each ESCRT can have progenitor-specific and lineage-restricted effects mediated by differential regulation of intra- and intercellular signaling. Further, we find that ESCRT perturbation affects Notch mediated crystal cell differentiation. Our study provides a means to deconstruct the roles of ESCRT in spatiotemporal segregation of signaling, giving further insight into progenitor heterogeneity.

## RESULTS

### ESCRT components are mis-expressed in hematological malignancies

**Fig. 1.**
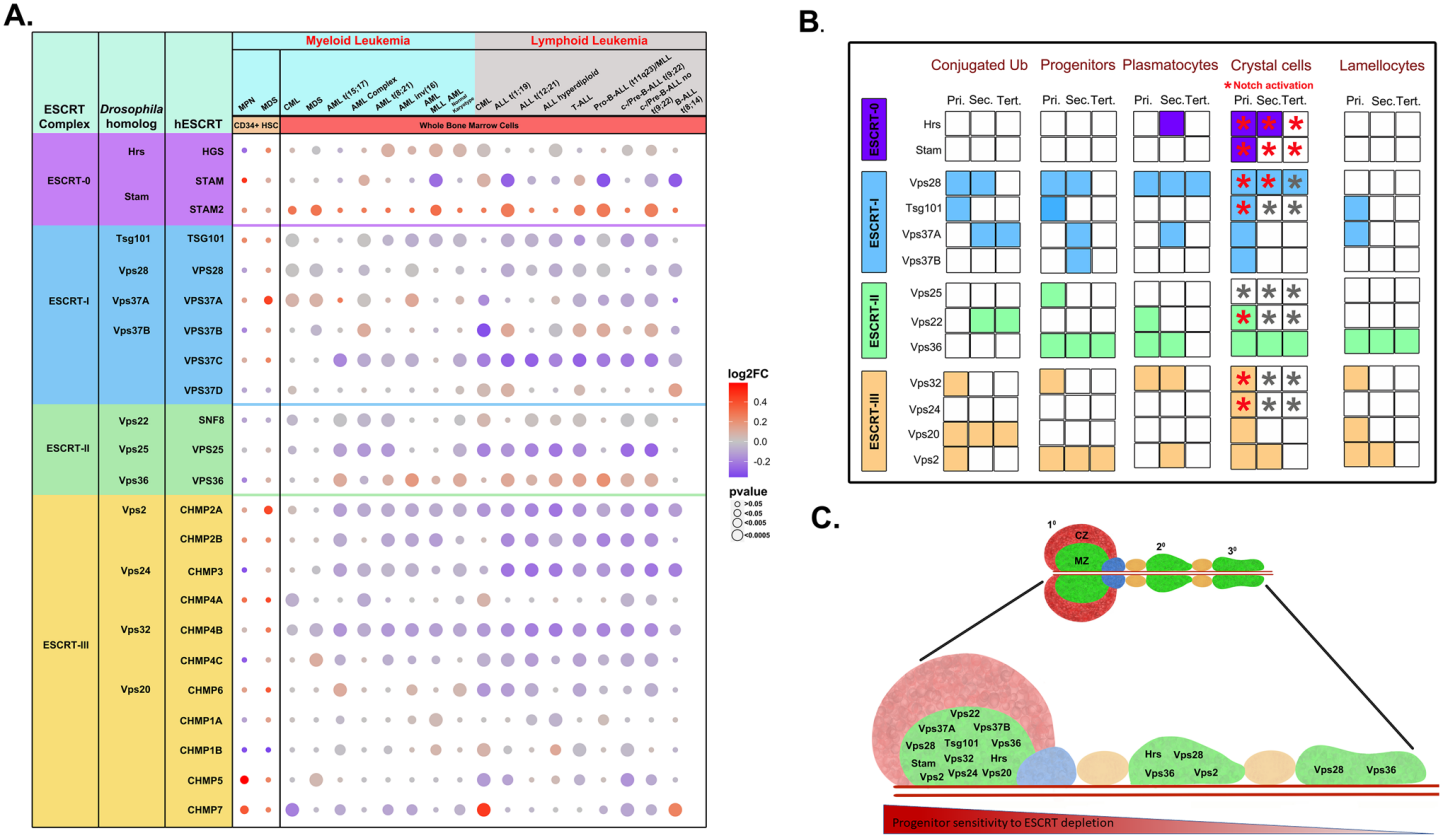
**Dysregulation of ESCRT components perturbs hematopoietic homeostasis.** (A) Change in expression levels of ESCRT components as found in CD34^+^ hematopoietic stem cells from MPN (Baumeister et al., 2021) and MDS patients (Pellagatti et al., 2010) as well as in whole marrow cells obtained from leukemia patients (BloodSpot, MILE study) is visualized using a bubble plot, across all classes of leukemia described in the study. Shades of red indicate overexpression while shades of blue indicate downregulation of individual components. The intensity of color is correlative to the log2 fold change while the size of the individual bubble indicates the *P*-value. The left-most column lists the *Drosophila* homologs of the human ESCRT proteins. (B) Comprehensive summary chart of the effects of individual ESCRT depletion on the various aspects of hematopoiesis as indicated. Presence or absence of a phenotype is depicted by colored or white boxes, respectively. Red asterisk indicates Notch pathway activation and empty asterisk indicates no change, for components that were tested. (C) Schematic representation of lymph gland lobes from anterior to posterior (left to right). Green region (medullary zone) in primary and posterior lobes indicate the progenitor pool depleted of ESCRT components (13 genes from ESCRT-0, I, II and III). Red region (cortical zone) in the anterior lobe represents the mature hemocytes. ESCRT components mentioned in each lobe are those whose depletion affected crystal cell differentiation. Red monochrome heatmap indicates position-dependent progenitor sensitivity to depletion of ESCRT.

The microarray innovations in leukaemia (MILE) study is a collection of gene expression data from patients suffering from hematological malignancies. Mining available data from the MILE study using BloodSpot, we found a significant correlation between perturbed expression of ESCRT components and hematological disorders (Fig. 1A; Table S1). All 16 major classes of hematopoietic disorders were associated with misexpression of multiple core ESCRT components. Up- or downregulation of a given ESCRT component was seen fairly uniformly across all lymphoid leukemia analyzed, whereas it was sporadic in myeloid leukemia, suggesting that balanced hematopoiesis may require subtle control of various ESCRT components. Thus, lineage-specific roles of ESCRT components may be important for the maintenance of particular blood cell populations.

Data from the MILE study reflects the expression in a mixed population of bone marrow cells. Therefore, we also referred to individual microarray-based myelodysplastic syndrome (MDS) (Pellagatti et al., 2010; Gorombei et al., 2021) and myeloproliferative neoplasm (MPN) (Baumeister et al., 2021) studies done for CD34^+^ hematopoietic stem cells (HSCs) to understand whether ESCRT expression is perturbed in stem and progenitor cells. ESCRT-III components CHMP5 and CHMP7 were overexpressed in MPN whereas Vps37A of ESCRT-I and Vps2 of ESCRT-III in MDS (Fig. 1A; Table S1). This suggests ESCRT-III may be more critical in the context of HSC maintenance and differentiation.

### ESCRT perturbation has non-uniform effects on ubiquitinated cargo sorting in the lymph gland progenitors

To test whether all ESCRT components have similar roles in cargo sorting in blood progenitors, we depleted each of the 13 core components individually in the lymph gland by RNAi-mediated knockdown (KD) using the domeless (dome)-GAL4 driver that expresses in blood progenitors (*domeGal4*>*UAS ESCRT RNAi*). We checked expression of representative ESCRT components from each subunit by immunofluorescence (IF) or RNA *in situ* hybridization in wild-type lymph gland (Fig. S1A-C). ESCRT components showed uniform expression across different lobes and developmental zones of the lymph gland. Next, we validated the knockdown of the ESCRT genes using immunofluorescence analysis, *in situ* hybridization or RT-qPCR (Fig. S1D-L).

As ESCRT plays an active role in ubiquitinated cargo sorting in multiple tissue and cell types, the accumulation of ubiquitinated cargoes serves as a hallmark of dysfunctional ESCRT machinery and impaired endosomal protein sorting. Immunofluorescence analysis of conjugated ubiquitin (Ub) status (see Materials and Methods) across all progenitor subsets (primary, secondary and tertiary lobes) showed a range of effects with the phenotype varying among ESCRT components within a given ESCRT complex and between complexes (Fig. 1B; Fig. S2).

Of the 13 core ESCRT components, seven caused increased Ub in the LG when depleted (Fig. 1B, Fig. S2). Interestingly, the effects were not uniform amongst progenitor subsets: five affected Ub status in the primary lobes, four in the secondary lobes and three in the tertiary lobes. Control LG showed low or no Ub in primary, secondary, and tertiary lobes. A similar trend was seen on depletion of ESCRT-0 components Hrs or Stam, with an occasional increase in Ub in primary lobes, which was not statistically significant (Fig. S2A,B). In contrast, ESCRT-I, -II and -III depletion had effects on all lobes, though not all components affected the Ub status. Among ESCRT-I components (Vps28, Tsg101, Vps37A, Vps37B), depletion of Vps28 or Tsg101 very significantly increased Ub in the primary lobe, Vps28 and Vps37A affected the secondary lobe and Vps37A showed an increase in Ub in the tertiary lobe. Vps37B depletion had a non-significant effect with a mild trend of Ub accumulation in posterior lobes. ESCRT-II components Vps25, Vps22 and Vps36 had no effect on the primary lobe. However, Vps22 depletion caused a dramatic increase in Ub in the secondary and tertiary lobes, where Vps25 and Vps36 depletion had no effect. Finally, depletion of ESCRT-III components (Vps32, Vps20 and Vps2) caused a significant increase in Ub in the primary lobes whereas secondary and tertiary lobes were sensitive only to Vps20 depletion. Interestingly, we observed such varying phenotype across progenitors despite uniform expression of ESCRT components, indicating post-transcriptional regulation of ESCRT function across progenitor compartments. Thus, we charted the spatiotemporal relation of Ub status to ESCRT depletion in LG progenitor subsets. We next tested whether this correlation reflects the response of progenitors to maintenance and differentiation cues and the developmental signaling pathways involved.

### ESCRT components play distinct roles in lymph gland progenitor maintenance

As ESCRT components regulate ubiquitinated cargo sorting in the blood progenitors, they might potentially regulate progenitor homeostasis. Hence, we checked whether depleting the 13 ESCRT components individually from dome^+^ progenitor subsets (marked by GFP expression) (*domeGal4>2XEGFP/+; UAS ESCRT-RNAi/+; +/+*) may affect progenitor status similar to the effect on Ub accumulation. As in the case of Ub status, a comprehensive analysis of progenitor fraction (see Materials and Methods) in the primary, secondary, and tertiary lobes of the lymph gland showed a range of effects with the phenotype varying among ESCRT components within a complex and between complexes (Fig. 1B; Fig. S3). In controls, anterior lymph gland lobe progenitors are restricted to the inner medullary zone (MZ) while the posterior lobes are composed almost entirely of progenitors (Rodrigues et al., 2021a). ESCRT-0 (Hrs, Stam) knockdown did not show any significant change in progenitor status indicating non-essential roles for these in progenitor maintenance (Fig. 1B; Fig. S3A,B). Depletion of ESCRT-I components Vps28 and Tsg101 caused reduction in progenitor fraction in the primary lobes whereas secondary lobe progenitors were reduced by depletion of Vps28, Vps37A or Vps37B but not of Tsg101. Interestingly, ESCRT-I components had no effect on tertiary lobe progenitors. Among ESCRT-II components, Vps25 depletion caused decrease in progenitor fraction (Fig. 1B; Fig. S3A,B). Vps22 also did not affect LG progenitor fraction. In contrast, Vps36 drastically reduced progenitor fraction in all LG lobes, with phenotype severity increasing from anterior to posterior. ESCRT-III had very restricted effects on progenitors with Vps32 KD causing a reduction only in anterior progenitors, Vps2 KD reduced both anterior and posterior progenitors and Vps24 and Vps20 had no effect (Fig. S3A,B).

Since increased Ub accumulation indicates dysfunctional cargo sorting and this is likely a cause of progenitor loss, we compared Ub status on ESCRT KD with progenitor maintenance and found limited correlation. While knockdown of some ESCRT components [(Vps28, Tsg101 (ESCRT-I), Vps32 and Vps2 (ESCRT-III)] caused increased Ub and reduced progenitors in the primary lobe, others [(Vps25, Vps36 (ESCRT-II)] showed no change in Ub but progenitor numbers were reduced. Similarly, knockdown of Vps20 (ESCRT-III) had increased Ub but no effect on progenitors. Hence, in addition to Ub cargo sorting, ESCRT components Vps25 and Vps36 have a critical independent role in progenitor maintenance. Notably, there was no correlation at all between Ub increase and progenitor reduction in the youngest progenitors (tertiary lobe). This suggests that notwithstanding defects in cargo sorting, posterior progenitors remain less sensitive to perturbation, suggesting that they are maintained by other robust mechanisms (Fig. 1C).

### Older progenitors are more prone to plasmatocyte differentiation upon ESCRT depletion

Plasmatocytes, marked by P1 expression, make up about 95% of the differentiated hemocyte population. In the LG, they are restricted to the cortical zone of the primary lobe, with occasional P1 positive cells seen in posterior lobes. Reduced progenitor numbers are expected to be accompanied by an increase in the plasmatocyte population due to differentiation. Enumeration of P1 positive cells in the ESCRT KD LG (*domeGal4 UAS 2XEGFP> UAS ESCRT RNAi*) showed an expected increase in the plasmatocyte fraction of the primary lobe for Vps28, Tsg101, Vps36 and Vps32, where the progenitor fraction was mostly reduced (Fig. 1B; Fig. S3A,C). However, Vps25 depletion had no apparent effect on differentiation. This could be due to the failure of progenitors to terminally differentiate into plasmatocytes or due to non-autonomous over-proliferation of the intermediate population. Additionally, Vps22 KD also showed increased plasmatocyte numbers though there was no significant effect on the progenitor fraction, suggesting possible non-autonomous over-proliferation or exhaustion of the intermediate population. The remaining ESCRT components had no effect on primary lobe plasmatocytes.

Interestingly, knockdown of ESCRT-0 component Hrs and ESCRT-III component Vps32, which had no effect on progenitors, caused an increase in plasmatocyte numbers only in the secondary lobes. This indicates that though there is no effect as assessed by progenitor marker analysis, Hrs or Vps32 depletion has sensitized the tissue to respond to proliferation and differentiation cues. Along similar lines, Vps36 and Vps2 depletion drastically reduced the secondary progenitor fraction and increased plasmatocyte differentiation. Except for Vps28, ESCRT KD did not induce plasmatocytes in the tertiary lobes, even when progenitors were lost (e.g. Vps36 KD and Vps2 KD).

### ESCRT components play distinct roles in regulating mitotic potential across different blood progenitor subsets

Altered cell proliferation can contribute to perturbed tissue homeostasis. Our analysis of blood cell differentiation is based on the estimation of the cell fraction, which could be an outcome of not only progenitor differentiation but also proliferation of individual blood cell types. ESCRT genes act as tumor suppressors in epithelial tissues by inhibiting Notch-dependent hyperplastic and neoplastic overgrowth (Hariharan and Bilder, 2006; Vaccari and Bilder, 2005; Horner et al., 2018). We tested whether downregulation of ESCRT expression can impact proliferation of blood cells. Analysis of eight selected ESCRT components showed that progenitor-specific knockdown of three ESCRT components [Vps28 (ESCRT-I), Vps22 (ESCRT-II) and Vps32 (ESCRT-III)] led to an increase in the number of nuclei with high mitotic potential in the primary lobe as revealed by high levels of phosphorylated Histone H3 (Fig. S4A,B). Vps32 knockdown also caused an increase in the size of the primary lobe as interpreted by the number of nuclei (Fig. S4A,C). However, Vps28 and Vps22 knockdown did not affect the overall size of the primary lobe. This suggests that cells may not have actively divided in the Vps28 and Vps22 depleted primary lobes despite the increase in the mitotic potential. Though Tsg101 and Vps25 knockdown did not increase the number of H3P high nuclei in the primary lobe, the overall size of the primary lobe increased. This suggests possible early developmental stage-specific effects on cell proliferation due to ESCRT depletion. Also, high proliferation may explain why Vps25 knockdown causes reduction in progenitor fraction without any significant change in plasmatocyte fraction (See Fig. 1B; Fig. S3A-C).

Depletion of Vps22 (ESCRT-II) resulted in increase in the mitotic potential in the secondary lobes (Fig. S4A,B). Depletion of six components [Hrs, Stam (ESCRT-0); Vps28, Tsg101 (ESCRT-I); Vps22 (ESCRT-II) and Vps32 (ESCRT-III)] however resulted in an increase in cell numbers in the secondary lobes (Fig. S4A,C). This, again, reflects temporal regulation of mitotic potential and cell proliferation upon knockdown of various ESCRT components. On the other hand, Vps25 (ESCRT-II) and Vps24 (ESCRT-III) knockdown reduced mitotic potential in the secondary lobe though the overall size of the secondary lobe remained unaffected (Fig. S4A-C). This indicates positive regulation of mitotic potential by Vps25 and Vps24 in the secondary lobe.

None of the ESCRT KD showed increased mitotic potential in the tertiary lobe. Rather, proliferative potential decreased in the tertiary lobe upon knockdown of four components [Vps28, Tsg101 (ESCRT-I); Vps25 (ESCRT-II) and Vps24 (ESCRT-III)] (Fig. S4A, B). However, tertiary lobe size increased upon knockdown of six components [Hrs, (ESCRT-0); Vps28 and Tsg101 (ESCRT-I); Vps22 (ESCRT-II); Vps32 and Vps24 (ESCRT-III)] (Fig. S4A, C) suggesting hyperplasia. In summary, depletion of ESCRT components promote proliferation but in a developmentally regulated manner.

Increased differentiation or proliferation can cause disintegration of the primary lobe or appearance of tumorous bulges resembling neoplastic overgrowth. To assess the change in morphology of the lymph gland lobes upon depletion of ESCRT components, we categorized lymph glands into three groups based on the appearance of lobe margin: regular, irregular/disintegrated, and tumorous bulge. While the majority of the control lymph gland primary lobes showed a regular boundary, knockdown of six ESCRT components [Hrs (ESCRT-0); Vps28, Tsg101 (ESCRT-I); Vps25, Vps22 (ESCRT-II) and Vps32 (ESCRT-III)] resulted in tumorous outgrowth in the primary lobe (Fig. S5A,B). Also, Hrs and Vps28 knockdown resulted in a significant increase in primary lobe disintegration as revealed by the irregular boundary. Vps28 and Vps32 knockdown resulted in significant increase in tumorous overgrowth in the secondary and tertiary lobe. Our analyses show that altered mitotic potential and cellular proliferation due to ESCRT depletion can contribute to altered blood cell homeostasis.

### ESCRTs downregulate Notch signaling to prevent crystal cell differentiation

As described earlier, eight of the 13 core ESCRT components affect progenitor numbers and these effects are position-dependent. To examine the effect of progenitor status on hemocyte differentiation, we assessed plasmatocytes, crystal cells and lamellocytes in the lymph gland. Plasmatocytes, marked by P1 expression, make up about 95% of the differentiated hemocyte population but their numbers were increased only in a few cases upon ESCRT depletion (Fig. 1B; Fig. S3). Under steady state conditions each primary lobe harbors approximately 0-10 crystal cells, while they are generally absent from posterior lobes, even upon immune challenge (Rodrigues et al., 2021a). Hence, we next analyzed crystal cell status by checking expression of ProPO in the ESCRT depleted LGs.

Knockdown of 12 out of 13 ESCRTs increased crystal cell differentiation in the primary lobes (Fig. 1B,C; Fig. S6), whereas Vps25 depletion had no effect. Secondary lobes were sensitive to depletion of Hrs, Vps28, Vps36 and Vps2 showing increased crystal cell numbers in all cases. Tertiary lobes showed increase in crystal cell numbers only on Vps28 or Vps36 depletion. This suggested that perturbation in the ESCRT machinery results in activation of signaling pathways that promote crystal cells. Notch pathway activation is a key requirement of crystal cell differentiation and Notch is a well-known target of ESCRT-mediated cargo sorting (Duvic et al., 2002; Lebestky et al., 2003; Vaccari and Bilder, 2005; Herz et al., 2009). Therefore, we examined the status of Notch pathway activation upon ESCRT depletion.

**Fig. 2.**
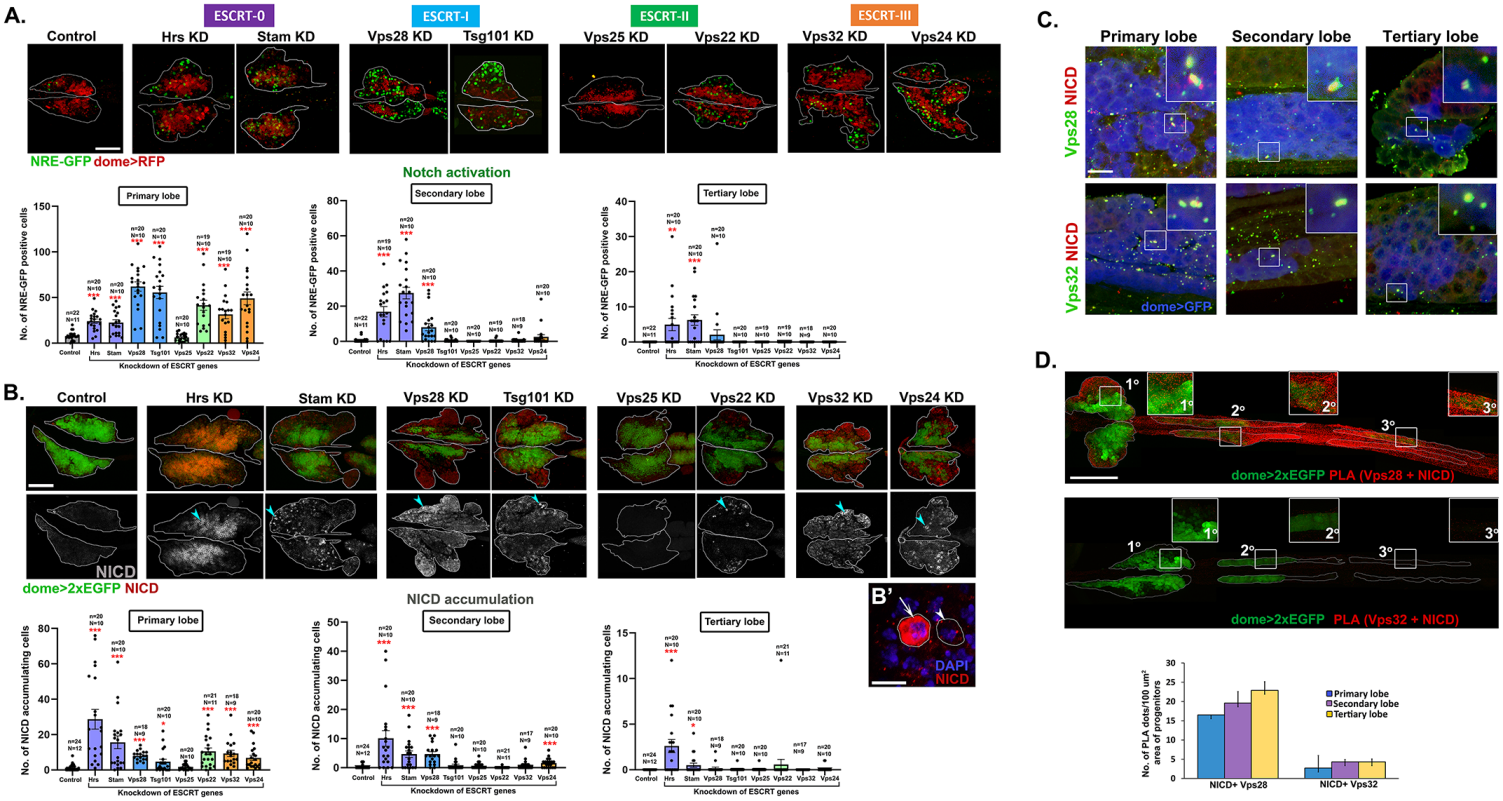
**ESCRT components regulate Notch activation and NICD trafficking in the lymph gland.** (A) Whole-mount larval lymph gland showing NRE-GFP+ve (Notch reporter) cells (green) and dome+ve progenitors (red) in primary lobes upon progenitor-specific knockdown of eight representative ESCRT components indicated [Hrs, Stam (ESCRT-0); Vps28, Tsg101 (ESCRT-I); Vps25, Vps22 (ESCRT-II); Vps32, Vps24 (ESCRT-III)]. Scale bar: 100 µm. Bar diagrams show quantification of the number of NRE-GFP positive cells in primary, secondary and tertiary lobes upon knockdown of the same eight ESCRT components. (B) Whole-mount larval lymph gland showing NICD expression (shown in red in the upper panel and in gray scale in the lower panel) in primary lobes upon progenitor-specific knockdown of the same eight aforementioned ESCRT components. Progenitors are marked by dome>2xEGFP (green). Scale bar: 100 µm. (B′) Magnified view showing lymph gland hemocytes with (arrow) or without (arrowhead) NICD accumulation. Scale bar: 10 µm. One-way ANOVA was performed to determine statistical significance. *P<0.05, **P<0.01, ***P<0.001. (C) Immunostaining for NICD (red) and ESCRT components Vps28 and Vps32 (green) showing colocalization in dome+ve progenitors (blue) across primary, secondary and tertiary lobes of the lymph gland. Scale bar: 10 μm. (D) PLA dots (red) mark the interaction of NICD with Vps28 and Vps32 across three lobes of the lymph gland. Progenitors are marked by dome>2×EGFP. Insets show a magnified view of the progenitors from primary, secondary and tertiary lobes. Scale bar: 200 μm. Bar diagram shows quantification of the number of PLA dots per 100 μm^2^ area of the progenitors. *N*=5 larvae.

Increased crystal cell differentiation upon depletion of some ESCRT components suggests that they normally suppress Notch pathway activation. We focused our study on analysis of eight ESCRT components [Hrs, Stam (ESCRT-0); Vps28, Tsg101 (ESCRT-I); Vps25, Vps22 (ESCRT-II); Vps32, Vps24 (ESCRT-III)] as these are well known for their role in NICD trafficking and Notch pathway activation (Vaccari et al., 2009; Tognon et al., 2014). The Notch response element driving GFP (NRE-GFP) is a useful reporter to assess activation of Notch signaling. Upon knockdown in the lymph gland progenitors, all components tested caused upregulation of Notch signaling as interpreted by an increase in NRE-GFP positive cells in the primary lobe, except Vps25 (Fig. 2A). Hrs, Stam and Vps28 depletion increased Notch activation in the posterior lobes (Fig. 2A, Fig. S7A). This is in concordance with the crystal cell differentiation phenotype as all of the ESCRT components except Vps25 cause increased crystal cell differentiation in the primary lobe upon knockdown. Our result also indicates that these seven ESCRT components are indispensable for regulation of Notch signaling in the blood cell progenitors possibly with non-compensatory roles.

NICD transport and cleavage is required for the activation of Notch signaling. Accumulation of NICD may lead to aberrant activation of Notch signaling. Progenitor-specific knockdown of all tested ESCRT components, except Vps25, resulted in increase in the number of cells accumulating NICD in the primary lobe (Fig. 2B). Also, this suggests a role for the majority of the ESCRT components in NICD trafficking, which may affect Notch signaling. However, the effects of perturbed Notch signaling on crystal cell differentiation vary in the posterior lobes, indicating position-dependent regulation. The absence of any phenotype due to Vps25 knockdown suggests that compensatory mechanisms may regulate cargo trafficking and lineage-specific signaling activation, which is sufficient to maintain progenitors at steady state.

### Position-dependent regulation of Notch signaling

Three out of the eight ESCRT components tested [Hrs, Stam (ESCRT-0) and Vps28 (ESCRT-I)], upon knockdown caused increased Notch signaling activation in the secondary lobe (Figs 1B and 2A; Fig. S7A). Of these only Hrs and Vps28 knockdown resulted in increased crystal cell differentiation in the secondary lobe (Fig. 1B,C; Fig. S6), suggesting additional mechanisms downstream of Notch activation prevent crystal cell differentiation in Stam KD. Interestingly, in the tertiary lobe, both Hrs or Stam knockdown resulted in Notch activation (Fig. 2A; Fig. S7A) though neither resulted in crystal cell differentiation (Fig. S6). In either case, tertiary lobe progenitors fail to differentiate, indicating their immature and refractile nature (Fig. 1B,C; Fig. S6). Also, though Vps28 depletion does not significantly activate Notch signaling in the tertiary lobe it can promote crystal cell differentiation, suggesting a possible mechanism to downregulate Notch signaling likely after progenitors differentiate. However, we do see occasional increase in Notch activation in Vps28 KD tertiary lobes. Hence, crystal cell differentiation in the tertiary lobe could be under complex temporal regulation. Our analysis demonstrates the active role of ESCRT components in regulating Notch signaling, which may contribute to crystal cell differentiation and also the differential response of the progenitor subsets.

### ESCRT components uniformly interact with NICD across progenitor subsets

As progenitor subsets respond differentially upon ESCRT depletion, we investigated whether co-expressing ESCRT components may differentially interact with endosomal cargoes in these different progenitor subpopulations. We chose two representative components, Vps28 and Vps32 that differentially regulate cargo sorting and progenitor homeostasis in the lymph gland. While Vps28 knockdown results in cargo accumulation and signaling activation in anterior as well as posterior subsets of progenitors, Vps32 depletion primarily affects anterior progenitor homeostasis. We performed both immunolocalization analysis and proximity ligation assay (PLA) of indicated ESCRT components with NICD since appropriate antibodies were available (Fig. 2C,D). Immunostaining-based analysis showed co-localization of Vps28 and Vps32 with NICD across all progenitor subsets (Fig. 2C). Using PLA to assess physical interaction, we found that while NICD interacted with Vps28 in progenitors of all three lobes as expected, direct physical interaction was negligible between Vps32 and NICD and did not vary across lobes (Fig. 2D). Our results suggest that the phenotypic diversity across lobes may not arise due to differential expression or subcellular localization of individual ESCRT. Additional regulators may cause differential sorting efficiency by ESCRT components across progenitor subsets and merit further exploration.

### Notch regulation downstream of ESCRT is independent of Notch ubiquitination regulators

**Fig. 3.**
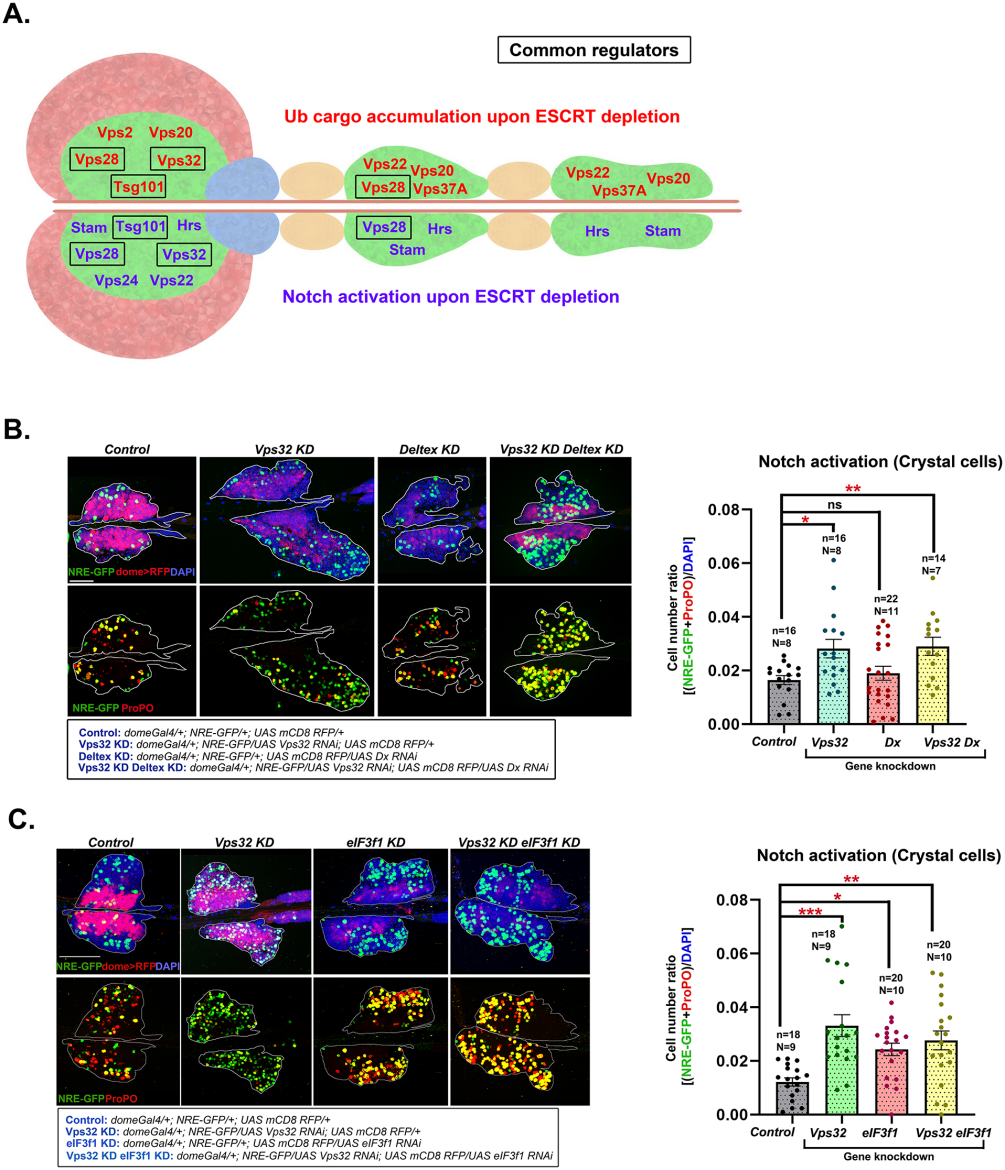
**Non-canonical ESCRT functions may be at play for maintenance of hematopoietic homeostasis.** (A) Schematic showing ESCRT components that affect ubiquitinated cargo sorting upon their depletion (in red) in the upper half and components that affect notch activation upon their depletion (in purple) in the lower half of the lymph gland schematic, in the respective lymph gland lobes. Black boxes indicate ESCRT proteins that affect both the processes in the respective lymph gland lobes. (B) Whole-mount larval lymph gland showing NRE-GFP and ProPO staining mark Notch activation and crystal cell differentiation, respectively in the primary lobe of control, Vps32 KD, eIF3f1 KD and Vps32 KD eIF3f1 KD lymph gland. Detailed genotypes are mentioned below the image panel of C and D. Bar diagrams show quantification of the fraction of NRE-GFP and ProPO positive cells in the primary lobe. n indicates the number of individual lobes analyzed and N indicates the number of larvae analyzed. Error bars represent s.e.m.; one-way ANOVA was performed to determine the statistical significance. *P<0.05, **P<0.01.

Knockdown of four components [Hrs, Stam (ESCRT-0), Vps28 (ESCRT-I) and Vps24 (ESCRT-III)] led to an increase in the number of NICD accumulating cells in the secondary lobe (Fig. 2B; Fig. S7B). However, only Hrs and Stam knockdown resulted in NICD accumulation in the tertiary lobe. This is in concordance with the phenotype of Notch activation upon Hrs, Stam and Vps28 knockdown in the posterior lobes. Our results show that NICD accumulation and Notch pathway activation correlate perfectly with crystal cell differentiation upon ESCRT knockdown. However, the status of ubiquitinated cargo accumulation does not correlate strongly with crystal cell differentiation phenotype (Fig. 3A). We hypothesized that Notch activation, triggered by ESCRT depletion in blood progenitors may be independent of the status of Notch ubiquitination.

Several ubiquitin ligases and deubiquitinases regulate Notch activation in various contexts (Moretti et al., 2010). Ubiquitin ligases such as Mindbomb, Neuralized, d-cbl, and Archipelago ubiquitinate the Notch-specific ligand Delta (Lai et al., 2001; Itoh et al., 2003; Wang et al., 2010). The Notch-specific E3 ubiquitin ligase Deltex positively regulates Notch pathway in a ligand-independent manner (Diederich et al., 1994). Also, Deltex acts in synergy with ESCRT-III component Vps32 in the *Drosophila* wing disc to fine-tune Notch signaling (Hori et al., 2011). The Notch-specific deubiquitinase eIF3f1 acts downstream of Deltex and promotes γ-secretase-dependent cleavage of NICD from the endosome surface, thus upregulating Notch signaling (Moretti et al., 2010). As both Deltex and eIF3f1 positively regulate Notch signaling in *Drosophila* tissues by directly regulating the ubiquitination or deubiquitination of Notch (Matsuno et al., 1995; Moretti et al., 2010; Hori et al., 2011), we probed into any possible genetic interaction between ESCRT and Deltex or eIF31 in blood progenitors, which may regulate Notch signaling.

Vps32 knockdown in the progenitors upregulates Notch signaling and crystal cell differentiation. However, progenitor-specific knockdown of Deltex (*domeGal4/+; UAS Vps32 RNAi/NRE-GFP; UAS Dx RNAi/UAS mCD8 RFP*) (Fig. 3B) or eIF3f1 (*domeGal4/+; UAS Vps32 RNAi/NRE-GFP; UAS eIF3f1 RNAi/UAS mCD8 RFP*) (Fig. 3C) failed to rescue the phenotype of Vps32 knockdown (*domeGal4/+; UAS Vps32 RNAi/NRE-GFP; +/UAS mCD8 RFP*). Deltex or eIF3f1 knockdown maintained high Notch activation in Vps32 knockdown lymph glands. Our result indicates that Notch regulation downstream of ESCRT is independent of Notch ubiquitination regulators, Deltex or eIF3f1, at least for the ESCRTs tested. This might explain why depletion of some ESCRT components, despite showing no significant accumulation of cargo in ubiquitinated state can promote Notch signaling and crystal cell differentiation.

### ESCRT depletion sensitizes progenitors for lamellocyte differentiation but does not confer survival advantage

Lamellocyte differentiation rarely occurs in the larva without any wasp infestation. However, progenitor-specific knockdown of six ESCRT components [Tsg101 and Vps37A (ESCRT-I); Vps36 (ESCRT-II); Vps32, Vps20 and Vps2 (ESCRT-III)] induced lamellocyte differentiation in the primary lobe, as visualized by Phalloidin (F-actin) staining, without any immune challenge (Fig. 1B; Fig. S9). Knockdown of two components (Vps36 and Vps2) resulted in lamellocyte differentiation in the secondary lobe and only Vps36 knockdown triggered lamellocyte differentiation in the tertiary lobe. This indicates that the majority of the ESCRT components are not involved in suppressing lamellocyte differentiation in the refractile posterior progenitors at steady state. However, it is likely that KD progenitors may be more sensitive to immunogenic cues as compared to normal, unperturbed progenitors.

**Fig. 4.**
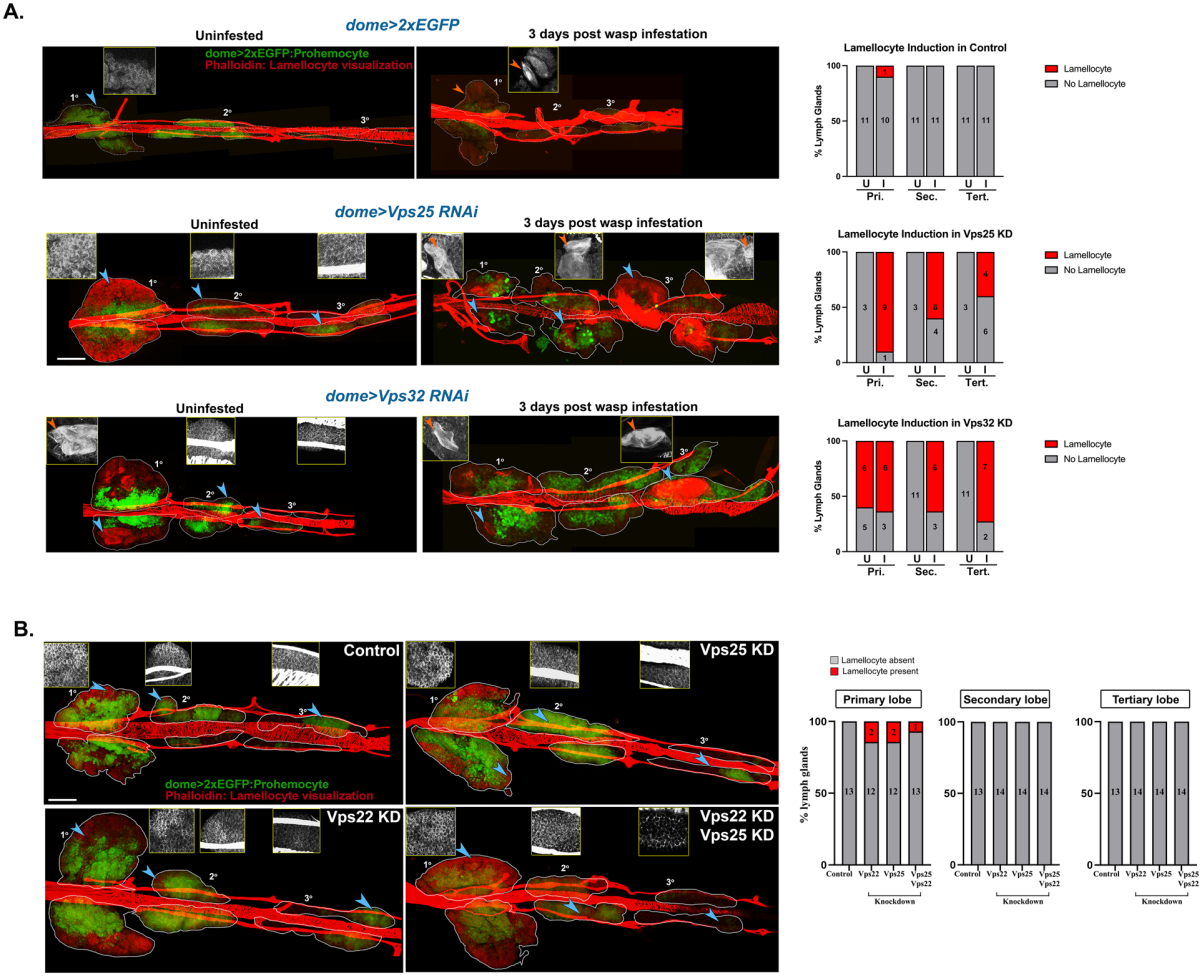
**ESCRT components are non-compensatory in function and regulate prohemocyte sensitivity to immunological cues.** (A) Whole-mount lymph glands of Vps25 KD (*domeGal4 UAS 2xEGFP; UAS Vps25 RNAi; +/+*) and Vps32 KD (*domeGal4 UAS 2xEGFP; UAS Vps32 RNAi; +/+*) larvae uninfested or 3 days after wasp infestation. Phalloidin staining shows presence of lamellocytes (marked by orange arrowhead in the inset). Bar diagram shows quantification of the percentage of lymph glands showing lamellocyte differentiation in primary, secondary and tertiary lobes upon knockdown of ESCRT components (Vps25 and Vps32), with and without immune challenge. Values in the columns indicate the number of larvae analyzed for presence or absence of lamellocytes. (B) Whole-mount larval lymph gland showing Phalloidin staining (red) to visualize elongated morphology of lamellocytes upon progenitor-specific knockdown of ESCRT-II components [Vps22 (*domeGal4 UAS 2xEGFP; UAS Vps22 RNAi; +/+*), Vps25 (*domeGal4 UAS 2xEGFP; UAS Vps25 RNAi; +/+*) and both Vps25 and Vps22 together (*domeGal4 UAS 2xEGFP; UAS Vps25 RNAi/ UAS Vps22 RNAi; +/+*)]. Blue arrowheads mark the region from primary, secondary or tertiary lobes, magnified in the insets. The inset panel shows enlarged view of Phalloidin staining with or without lamellocytes. Scale bar: 100 µm. Bar diagram shows quantification of the percentage of lymph glands showing lamellocyte differentiation in primary, secondary and tertiary lobes upon knockdown of ESCRT-II components, without any immune challenge. Values in the columns indicate the number of larvae analyzed for presence or absence of lamellocytes.

Wild-type larvae are generally able to mount a sufficiently robust immune response that aids in their survival and eclosion against a low dose of wasp infestation. Systemic signals are generated upon wasp infestation and are received by the lymph gland progenitors (Morin-Poulard et al., 2013), possibly through a complex extracellular matrix (Rodrigues et al., 2021a). This results in lamellocyte differentiation in the primary lobe followed by disintegration and release of lamellocytes into circulation. Secondary and tertiary lobes are refractile to wasp infestation and do not form lamellocytes even upon immune challenge. Hence, we chose to test lamellocyte differentiation upon wasp infestation in: (a) ESCRT KD that had no effect on lamellocyte formation (Vps25 KD) and (b) ESCRT KD that caused lamellocyte differentiation only in the primary lobe (Vps32 KD). Knockdown of either Vps25 or Vps32 triggered lamellocyte differentiation across all progenitor subsets upon immune challenge with wasp (Fig. 4A). This shows that Vps25 and Vps32 play essential roles in preventing all posterior progenitors from lamellocyte differentiation in response to a natural immune challenge. Further, loss of ESCRT sensitizes progenitors to systemic cues by unlocking differentiation programs.

Next, we performed a developmental analysis in ESCRT knockdown flies with or without wasp challenge. Hemocytes are essential for normal pupariation and eclosion in *Drosophila* (Stephenson et al., 2022). Vps25 knockdown did not affect pupariation (control: 92.73%, Vps25 KD: 93.72%) but reduced eclosion (control: 93.58%, Vps25 KD: 88.13%) as well as survival after eclosion. To abrogate the possibility of neuron-specific effect of Vps25 depletion on development and survival, we additionally used elavGal80;;domeMESOGal4 driver to prevent Vps25 knockdown in the brain. This led to an increase in pupariation but showed only a mild effect on eclosion and led to reduced lifespan of adult flies, similar to domeGal4>Vps25 KD (pupariation – control: 73.33%, Vps25 KD: 93.68%; eclosion – control: 92.3%, Vps25: 95.28%) (Fig. S10B). Vps32 knockdown using domeGal4 showed normal pupariation, however, leading to death in the pupal stage (Fig. S10A). Suppressing knockdown in neurons using elavGal80;;domeMESOgal4 showed no effect on pupariation or eclosion and caused a mild reduction in adult fly survival between 30-60 days (Fig. S10B). domeGal4-driven knockdown of Vps36 leads to spontaneous differentiation to all lineages across progenitor subsets. However, it did not affect pupariation (control: 95.79%, Vps36 KD: 92.54%) or eclosion (control: 94.61%, Vps36 KD: 94.44%) (Fig. S10A). Our analyses show that altered hematopoiesis due to blood progenitor-specific depletion of ESCRT does not considerably affect post-embryonic development until eclosion, indicating normal function of the differentiated hemocytes.

To test whether increased differentiation provides any survival advantage, we assessed survival in wasp challenged Vps25 KD, Vps32 KD and Vps36 KD larvae (elavGal80;;domeMESOGal4-driven). ESCRT knockdown in the progenitor did not provide any survival advantage to the larvae as nearly all of the pupae failed to eclose with a lethal (moderate) dose of infection (survival: control 7.4%, Vps25 KD: 3%, Vps32 KD: 8.4%, Vps36 KD: 1.1%). Hence, *a priori* sensitization of progenitors does not promote survival upon immune challenge (Fig. S10C). This could be due to rapid exhaustion of the progenitors in the late larval stage and insufficiency of lamellocyte count required to combat infection despite increased lamellocyte differentiation in the lymph gland. It is also likely that ESCRT depletion compromises lamellocyte ability to complete one or more steps involved in encapsulation, given the large number of signaling pathways that depend on ESCRT.

### Blood progenitor homeostasis does not require functional compensation between dispensable ESCRT components from the same subunit

Analysis of lamellocyte differentiation reflects functional diversity across ESCRT components (see Fig. 1B; Fig. S9). Also, phenotypic diversity was observed within the same ESCRT subunit. Knockdown of ESCRT-II component Vps36 triggers spontaneous lamellocyte differentiation in steady state while depletion of the other two ESCRT-II components Vps25 or Vps22 does not affect lamellocyte differentiation. To confirm that Vps25 and Vps22 do not compensate each other in lamellocyte differentiation process, we generated larvae with double knockdown of Vps25 and Vps22 in the blood progenitor (*domeGal4 UAS2xEGFP/+; UAS Vps22 RNAi/UAS Vps25 RNAi;+/+*). Like the individual knockdown of Vps22 and Vps25, the double knockdown also did not trigger any significant lamellocyte differentiation under steady state (Fig. 4B). This indicates that Vps22 and Vps25 do not take part in regulating lamellocyte differentiation under steady state condition. Hence, absence of lamellocyte differentiation upon knockdown of certain ESCRT components is not due to functional compensation by other core components within the same ESCRT subunit. However, auxiliary components might functionally substitute core components in the blood progenitor which merit further investigation.

### ESCRT exhibits context dependent cell-autonomous and non-cell-autonomous roles in hematopoiesis

**Fig. 5.**
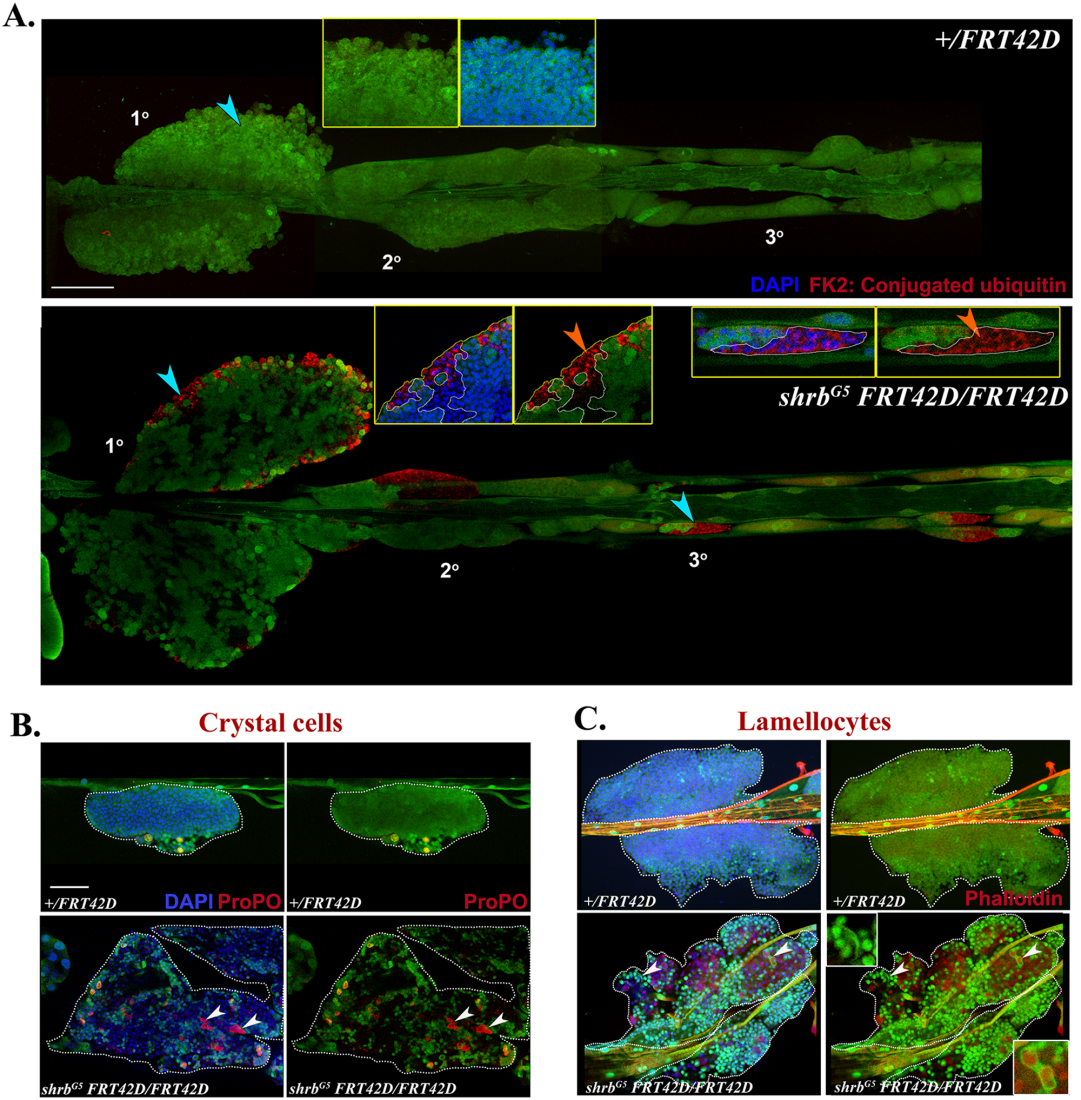
**ESCRT cell-autonomously regulates ubiquitinated cargo sorting in the lymph gland progenitors but may regulate differentiation in non-autonomous manner as well.** (A) Whole-mount lymph glands showing immunostaining for conjugated ubiquitin (red) in control (*domeGal4/+; neoFRT42D/+; UAS mCD8 RFP/+*) and progenitor-specific mutant clone of representative ESCRT component Vps32/shrub (*domeGal4/+; shrb^G5^ neoFRT42D/neoFRT42D; UAS mCD8 RFP/UAS FLP*). Area marked by blue arrowheads are magnified in the insets to show homozygous mitotic clones (GFP-ve patch, demarcated by dotted white line). Orange arrowheads indicate ubiquitin accumulation in the mutant cells. DAPI marks the nuclei. (B) Primary lobe of control and Vps32 mutant clone showing ProPO staining (red) to mark crystal cells. Arrowheads mark the crystal cells which are GFP-ve (homozygous mutant). (C) Phalloidin staining in the same genotypes shows GFP+ elongated and coalescing cells marked by arrowheads (insets) in the mutant clone. Scale bar in all image panels: 100 µm.

Progenitor-specific downregulation of ESCRT expression leads to accumulation of ubiquitinated cargoes. However, the majority of the ubiquitin accumulates in non-progenitor (Dome^−^) cells, suggesting a possible cell non-autonomous effect. The other possibility could be persistent ubiquitin accumulation even after the progenitors differentiate and lose Dome expression. We generated homozygous mutant mitotic recombinant clones for a representative ESCRT gene Vps32 (shrub), in progenitors. Vps32 is a terminally acting ESCRT (ESCRT-III) in the ubiquitinated cargo sorting pathway. Its depletion affects all blood cell lineages (Fig. 1B). Hence it serves as a good model to assess cell autonomous roles of endosomal protein sorting. Staining for conjugated ubiquitin revealed accumulation of ubiquitin aggregates in the homozygous mutant patch of cells in the cortical zone (GFP^−^) indicating accumulation of cargo in ESCRT-depleted blood progenitors (Fig. 5A). This suggests that despite a decrease in Dome expression, ubiquitinated cargo sorting defects can persist in the ESCRT depleted (knockdown or knockout) cells.

We analyzed differentiation of blood cells in mitotic clones of ESCRT. ProPO staining showed both wild type and mutant origin of crystal cells as revealed by overlap with GFP expression in the mutant tissue (Fig. 5B). As crystal cells are usually present in the lymph gland in low numbers, it is difficult to interpret the cell-autonomous origin of crystal cells from mutant progenitors. However, lamellocytes are completely absent in the control lymph gland at steady state (Fig. S9). Phalloidin staining in the Vps32 mutant clone showed GFP-expressing elongated or coalescing cells, indicating the presence of lamellocytes and possibly their precursors (Fig. 5C). This suggests non-autonomous regulation of lamellocyte differentiation by ESCRT. Hence, ESCRT may regulate progenitor differentiation in both a cell-autonomous as well as cell non-autonomous manner. Since cell non-autonomous effects might hinge on cell–cell communication either through junctions or juxtacrine signaling, it suggests an overarching effect of ESCRT depletion to bring about differentiation of neighboring cells to a particular lineage.

### Vps25 is dispensable for progenitor maintenance

Vps25 knockdown did not affect the status of ubiquitination, progenitor maintenance or differentiation to any particular blood cell lineage despite its expression in the lymph gland. To further verify this, we generated lymph gland progenitor-specific homozygous clones of Vps25 loss of function mutation (Vps25^A3^). There was no accumulation of ubiquitin aggregates or any change in the status of the progenitor (Fig. S8A), plasmatocyte (Fig. S8B), crystal cell (Fig. S8C) and lamellocyte differentiation (Fig. S8D). However, the mutant lymph glands showed enlargement of the primary lobe, suggesting possible increase in blood cell proliferation upon loss of Vps25. Also, phalloidin staining revealed appearance of binucleate, large cells and also very small cells occasionally, along with increase in F-actin content in some patches of the tissue, mostly in a cell autonomous manner (visible in GFP negative area of the tissue). Hence, Vps25 possibly inhibits uncontrolled cell proliferation and may contribute to critical steps of cell division that may dictate cell shape, number and polarity.

## DISCUSSION

Cargo sorting by the ESCRT machinery is ubiquitously essential. Earlier work from our laboratory has shown that endosomal cargo sorting defects caused by the depletion of a conserved regulator of hematopoiesis, Asrij, leads to precocious differentiation of blood progenitors. Mining available datasets of patient samples also indicated misexpression of ESCRT components in various hematological malignancies. Therefore, we asked whether ESCRTs play a decisive role in blood progenitor maintenance and lineage choice. Due to the pleiotropic subcellular roles of ESCRT proteins, it becomes challenging to correlate tissue phenotypes to specific ESCRT function (Stempels et al., 2023). Our study now provides a means to test the functional complexity of ESCRT using a simple yet comprehensive model of hematopoiesis, allowing in-depth *in vivo* analysis.

We show that endosomal protein sorting actively maintains hematopoietic homeostasis. We uncover specific steps of endosomal protein sorting that potentially dictate blood progenitor maintenance. ESCRT-I remodels the endosomal membrane through budding and ESCRT-III carries out scission, to allow cargo sorting. Loss of ESCRT-I or ESCRT-III components result in progenitor differentiation suggesting that membrane budding and scission affects a wide range of signaling pathways across distinct progenitor subsets. Recent reports highlight the universal role of ESCRT-III, often in concert with ESCRT-I, in various membrane remodeling processes such as nuclear envelope reformation, lysosomal membrane repair, cytokinetic abscission, macroautophagy, exocytosis and lipid transport to mitochondria via lipid droplets (Olmos et al., 2015; Wang et al., 2021). Whether such moonlighting functions of ESCRT-I and ESCRT-III impact signaling that determines progenitor maintenance merits further investigation.

Curiously, we observed drastic functional diversity of the highly conserved ESCRT-II components in progenitor differentiation. While Vps36 depletion affected all progenitor subsets, Vps25 depletion did not affect differentiation at steady state. Loss of Vps25 caused hyperproliferation in blood cells and failed to activate signaling pathways such as Notch, which are necessary for progenitor differentiation. Though Vps25 regulates endosomal protein sorting in epithelial tissues (Vaccari and Bilder, 2005; Herz et al., 2006), its redundancy in controlling differentiation suggests additional alternate routes for endosomal protein sorting in blood progenitors or a temporally regulated, developmental stage-specific role of Vps25 that has not yet been identified. Notably, though Vps25 is dispensable for steady state hematopoiesis, its depletion sensitizes all progenitors to differentiate upon immune challenge. This supports the possibility that the diverse roles of ESCRT may contribute to differential regulation of steady state and stress hematopoiesis.

Lymph gland progenitor subsets are functionally heterogeneous and show reduced sensitivity to differentiation cues from anterior to posterior (Rodrigues et al., 2021a; Ray et al., 2021). Posterior progenitors resist differentiation upon immune challenge suggesting that they have additional signal regulatory checkpoints. The mechanism by which progenitor subsets differentially respond to systemic cues remain largely unexplored. One possibility is that younger progenitors have inherently low levels of ubiquitination and protein turnover and hence show mild effects on ESCRT depletion. Depletion of Vps36 and Vps2 can trigger lamellocyte differentiation in refractile progenitors even without any immune challenge, indicating that they actively prevent differentiation. Also, while Hrs knockdown activates Notch signaling and crystal cell differentiation in posterior progenitors, Stam knockdown fails to trigger terminal differentiation to crystal cells despite Notch activation. This indicates existence of multiple checkpoints and highlights the complexity of mechanisms that progenitors may employ to maintain their identity. Elucidating expression and function at the single cell level may aid in an improved understanding of ESCRT-dependent lineage specification across these distinct progenitor subsets. Nevertheless, such candidates can be screened further for efficient modulation of vertebrate blood regeneration *in vitro* as well as *in vivo*.

Our study shows the distinct role of individual components of ESCRT in Notch signaling in blood progenitors. Expectedly, regulation of Notch signaling in lymph gland progenitors relies heavily on the ESCRT machinery. While activation of Notch promotes distal progenitor differentiation to crystal cells at the boundary of CZ and MZ, it may also promote progenitor maintenance at the MZ core (Blanco-Obregon et al., 2020; Ho et al., 2023). Here we show that ESCRT depletion in dome+ progenitors primarily promotes differentiation. Our previous reports highlight the potential functional link of endosomal protein sorting with blood progenitor homeostasis (Kulkarni et al., 2011; Khadilkar et al., 2014). Though Hrs and Stam depletion promoted crystal cell differentiation in the lymph gland, it hardly affected plasmatocyte and lamellocyte differentiation. Previous reports show that Hrs and Stam-dependent Notch trafficking does not regulate Notch pathway activation and downstream phenotypes such as cell polarity and proliferation (Tognon et al., 2014). This suggests that additional molecular players may regulate ESCRT-dependent phenotypes. Similar mechanisms specific to hematopoietic tissue are not yet explored. It is notable that ALIX and its yeast homolog Bro1 can recognize non-ubiquitinated cargoes and sort them independent of ESCRT-0 (Pashkova et al., 2013). Also, Bro1, ALIX and HD-PTP act as alternate bridging factors to ESCRT-II to mediate endosomal protein sorting in yeast and mammalian cells (Bissig and Gruenberg, 2014; Tabernero and Woodman, 2018). Post-translational regulatory mechanisms may also render ESCRT components inactive (Tsunematsu et al., 2010).

The role of ESCRT in lamellocyte differentiation indicates the impact on upstream signaling pathways. EGFR signaling promotes plasmatocyte proliferation and lamellocyte differentiation. Also, downregulation of JAK/STAT and Hedgehog signaling leads to progenitor loss and lamellocyte differentiation (Mandal et al., 2007; Morin-Poulard et al., 2013; Rodrigues et al., 2021a). While the role of ESCRT in EGFR and Hh signaling is known (Vaccari et al., 2009; Mattissek and Teis, 2014; Szymanska et al., 2018) the same in hematopoiesis is not dissected so far. Our analysis suggests that ESCRTs impact signaling pathways upstream of lamellocyte differentiation. Thus, we speculate that lineage-specific signaling activation could be achieved through modulation of specific ESCRT expression and function.

Deltex and eIF3f1 positively regulate Notch signaling in epithelial tissues through Notch ubiquitination (Matsuno et al., 1995; Moretti et al., 2010; Hori et al., 2012). However, downregulation of Dx/eIF3f1 failed to rescue Notch activation phenotype in ESCRT depleted progenitors. While other E3 ubiquitin ligases and deubiquitinases may possibly complement for Deltex or eIF3f1 depletion, signaling activation may not always depend on the status of cargo ubiquitination. For example, Vps36 depletion elicits a strong phenotype of differentiation without causing any change in ubiquitination. Further genetic interaction-based studies with other regulators of Notch signaling may reveal ESCRT-dependent mechanisms of Notch activation.

Loss of function mutation in ESCRT genes result in cell-autonomous cargo accumulation in *Drosophila* epithelial tissues (Vaccari et al., 2009; Herz et al., 2009). However, the known cell non-autonomous role of ESCRT in cell proliferation as well as neoplastic transformation, suggests altered intercellular communication and aberrant signaling activation in the neighboring cell population (Vaccari and Bilder, 2005; Vaccari et al., 2009; Herz et al., 2009). In concordance with the previous reports, we observed a cell-autonomous role of ESCRT in regulating ubiquitinated cargo sorting in the blood progenitors. However, analysis of lamellocyte differentiation suggests that ESCRT may affect lineage-specification non-autonomously. Both progenitor differentiation and proliferation influence blood cell homeostasis in the lymph gland. ESCRT depletion not only activates lineage-specific signaling pathways but also promotes blood cell proliferation. Cell type-specific increase in proliferation and enlargement of lymph gland lobes can affect the proportion of different hemocyte populations. Elucidating the interplay between ESCRT components and the mitogenic signaling machinery could reveal whether downregulation of mitotic potential may restore hematopoietic homeostasis.

Over 90% of hematological malignancies are associated with misexpression of one or more core ESCRT components. Hematological anomalies such as acute lymphoblastic leukemia (ALL), acute myeloid leukemia (AML), myelodysplastic syndromes, etc., stem from hyperproliferation, improper lineage choice and blood cell dysfunction caused by aberrant activation of signaling pathways. While reports show the importance of ESCRT in the hematopoietic and immune system (Adoro et al., 2017; Liu et al., 2021), any functional analysis relating to progenitor maintenance and fate choice remained underexplored. The phenotypic diversity we observe highlights the role of the conserved ESCRT machinery in blood progenitor maintenance and differentiation. This may have implication in understanding blood disorders and designing target-specific therapeutic interventions.

## MATERIALS AND METHODS

### Fly stocks and genetics

*Drosophila melanogaster* stocks were maintained at 25°C as described previously (Kulkarni et al., 2011). Canton-S was used as the wild-type reference strain. For progenitor-specific knockdown of ESCRT genes, *domeGal4 UAS-2xEGFP/FM7a* and *domeGal4/FM7b* (Utpal Banerjee, UCLA) were used as driver as well as parental control. *UAS-dsRNA* (RNAi) transgenic lines were used for the knockdown of Hrs (BDSC 33900), Stam (VDRC 22497), Vps28 (VDRC 31894), Tsg101 (BDSC 38306), Vps37A (BDSC 38304), Vps37B (BDSC 60416), Vps36 (BDSC 38286), Vps22 (BDSC 38289), Vps25 (VDRC 108105), Vps2 (BDSC 38995), Vps20 (Spyros Artavanis-Tsakonas, Harvard Medical School), Vps24 (BDSC 38281) and Vps32 (VDRC 106823). Other stocks used were *UAS mCD8 RFP* (BDSC 27399), NRE-GFP/CyO (BDSC 30727), *UAS dx RNAi* (BDSC 44455), *UAS eIF3f1 RNAi* (BDSC 33980), *UAS FLP* (BDSC 4540), *shrb^G5^ FRT42D/CyO GFP* (BDSC 39635), *FRT42D Ubi-GFP/CyO* (BDSC 5626), *Vps25^A3^ FRT42D/CyO GFP* (BDSC 39633). For developmental and survival analysis of some ESCRT components, elavGal80;;domeMESOGal4 (Tina Mukherjee, inStem) was used.

To obtain flies with prohemocyte-specific knockdown of target genes, homozygous RNAi male flies were crossed with domeGal4 UAS-2xEGFP/FM7a virgin females. F1 progenies were collected based on GFP expression. domeGal4 UAS-2xEGFP/FM7a was used as the parental control for all analyses. For experiments using *domeGal4/FM7b* driver, virgin females were crossed with UAS mCD8 RFP males. RFP positive female progenies were crossed with NRE-GFP male flies in F1 generation. GFP, RFP double positive flies were crossed with homozygous RNAi lines. GFP, RFP double positive F2 progenies were used for experiments and *domeGal4/+; NRE-GFP/+; UAS mCD8 RFP/+* larvae were used as parental control.

Progenitor-specific mosaic mitotic clones were generated using *domeGal4* driven recombination. *domeGal4; FRT42D Ubi-GFP; UAS mCD8 RFP* flies were used as controls. Genotype of the mitotic clones: (i) *domeGal4; shrb^G5^ FRT42D/FRT42D Ubi-GFP; UAS FLP/UAS mCD8 RFP*, (ii) *domeGal4; Vps25^A3^ FRT42D/FRT42D Ubi-GFP; UAS FLP/UAS mCD8 RFP*

### Immunostaining analysis

*Drosophila* third-instar larval lymph glands were dissected in PBS as described before and immunostained for microscopic analysis (Rodrigues et al., 2021b). Briefly, the dorsal half of the cuticle with brain lobes and cardiac tube intact, was fixed with 4% paraformaldehyde, washed in PBS, permeabilized in 0.3% Triton X-100, blocked with 20% normal goat serum and incubated overnight with the appropriate primary antibody dilutions. Primary antibodies used were guinea pig anti-Hrs (Benny Shilo, Weizmann Institute; 1:100), rabbit anti-dVps28 (Helmut Kramer, UT Southwestern Medical Center; 1:100), rabbit anti-Shrub (Fen B. Gao, University of Massachusetts, USA; 1:100), mouse anti-Tsg101 (ab83, Abcam, UK; 1:25), mouse anti-P1 and L1 (Istvan Ando, BRC Schezed, Hungary; 1:30), mouse anti-ProPO (1:20), FK2 (BML-PW8810-0100, Enzo Life Sciences, USA; 1:200), chick anti-GFP (ab13970, Abcam, UK; 1:200), rabbit anti-dsRed (632496, Takara, Japan; 1:200), mouse anti-NICD (C17.9C6c, DSHB, USA; 1:10), rabbit anti-phospho Histone H3 (06-570, Merck Millipore, USA; 1:200). Subsequently, samples were washed again and incubated in secondary antibodies, washed and mounted in DAPI-glycerol. For secondary antibody staining, Alexa Fluor conjugated anti-mouse, anti-rabbit or anti-guinea pig antibodies (Life Technologies, Thermo Fisher Scientific, USA) were used. Phalloidin conjugated to Alexa 568 or 633 (A12380, A22284, Life Technologies, Thermo Fisher Scientific, USA) was used to visualize lamellocytes. Images were acquired on Zeiss LSM880 Laser Confocal Microscope.

### MILE data analysis

For each ESCRT component, the expression values in each biological context (here, diseased and healthy bone marrow) were downloaded from BloodSpot (https://www.fobinf.com/). The expression values using all the probes for a particular gene were averaged and then the average expression values were normalized with the healthy bone marrow expression. Student's *t*-test was performed for expression of each ESCRT component in each disease with healthy bone marrow using the BloodSpot plugin, and the *P*-values were noted. For the CD34^+^ hematopoietic stem cells (HSC) from Myeloproliferative Neoplasm (MPN) (GSE174060) and Myelodysplastic Syndrome (MDS) (GSE19429) patient samples, data was sourced from Baumeister et al., 2021 and Pellagatti et al., 2010 respectively, using GEO (Baumeister et al., 2021, Pellagatti et al., 2010). The datasets were analyzed using GEO2R (LIMMA analysis with Benjamini-Hochberg FDR correction, adj. *P*-value <0.05). The log2 values of the obtained fold change were then plotted along with the *P*-value, in both cases, using the ggplot2 package in R. The resulting bubble plot indicates the log2FC values by the intensity of color, where red shows upregulation and blue downregulation, while the size of each bubble represents the *P*-value.

### *In situ* hybridization

RNA *in situ* hybridization was performed to check the expression of the ESCRT components against which antibodies are not available. The genes of interest (*Stam*, *Vps22*, *Vps25*, *Vps24*) were PCR amplified from genomic DNA using specific primer pairs as below, with the T7 promoter sequence incorporated in the reverse primer: *Stam* 5′ TTGTCACTGCCGATCTGTCC 3′ and 5′CCTGCTAATACGACTCACTATAGGGTTGACCCAGATAGCCACC 3′; *Vps22* 5′ACGTGATTTAGGTGACACTATAGTAGGCCTGGGAGCCATACAG 3′ and 5′ CCGTTAATACGACTCACTATAGGGTGCCAAAAATGCTCAATTTC 3′; *Vps25* 5′ CCCCAATTTAGGTGACACTATAGCGAAGAAACCAGACAGCAGC 3′ and 5′ TAATACGACTCACTATAGGGAAGAACTTAACGCCGTGGCTG 3′; *Vps24* 5′ GAGCCTGGTGCGCTATCC 3′ and 5′ GGCTTTAATACGACTCACTATAGGGTGCATCTCTTGCAGTTCCTC 3′

200ng-1 µg of the amplicon was subjected to *in vitro* transcription using DIG-labelling mix (Roche, Switzerland) to generate DIG labelled RNA probes. Length of the probes are 644 bp (Stam), 503 bp (Vps25), 500 bp (Vps22) and 325 bp (Vps24). *In situ* hybridization was performed as described in Benmimoun et al. (2015).

### RT qPCR

2-2.5 µg mRNA isolated from 100 lymph glands using Qiagen RNeasy kit was subjected to reverse transcription using Superscript (Invitrogen). 20 ng cDNA was used for each qPCR reaction of *Stam*, *Vps25, Vps24* and *Rp49*. All SYBR green- (Bio-Rad, USA) based experiments were performed in triplicates. Relative fold change was estimated by normalizing over Rp49. Primers used were *Stam* 5′ ACTGAAAATGCGCCAAGTGC 3′ and 5′ CGGCAACAGTCTTGCTAGTC 3′; *Vps25* 5′ CCCTTCTTTACACTACAGCC 3′ and 5′ CTGGTCCCCAATGCTGAGAG 3′; *Vps24* 5′ AAGAGCAGGTGCAGGAGTGG 3′and 5′ CAAGAATGACGCAGGTGTCG 3′; *Rp49* 5′ CCGCTTCAAGGGACAGTATC 3′ and 5′ ACA ATC TCC TTG CGC TTC TTG 3′.

### Developmental analysis

Ten virgin females were crossed with five males in each vial containing cornmeal medium supplemented with yeast. After 8-10 h of egg laying, the parents were flipped to a fresh vial for the next set. For each cross, 10 sets were made. The genotypes used were as follows:

With *domeGal4 2xEGFP* driver- control: *domeGal4, UAS-2xEGFP>/Fm7a; +; +,* Vps25 KD: *domeGal4, UAS-2xEGFP/+; UAS-Vps25 RNAi/+; +,* Vps36 KD: *domeGal4, UAS-2xEGFP/+ ; UAS Vps36 RNAi/+; +,* Vps32 KD: *domeGal4, UAS-2xEGFP/+; UAS-Vps32 RNAi/+; +*.

With Elav-Gal80;;Dome-mesoGFP> driver, control: elavGal80*; +;* domeMESOGFP>/+*,* Vps25 KD: elavGal80*; UAS-Vps25 RNAi*/+*;* domeMESOGFP>/+*,* Vps36 KD: elavGal80*; UAS Vps36 RNAi*/+*;* domeMESOGFP>/+*,* Vps32 KD: elavGal80; *UAS-Vps32 RNAi/+;* domeMESOGFP>/+.

Over 200 wandering third-instar larvae were collected and sorted on the basis of GFP expression from each cross and age matched larvae were kept together. They were observed for pupariation and the subsequently eclosed flies were collected and their life span was recorded. The collected flies were flipped every alternate day to fresh vials, to avoid bacterial or fungal infections. Records were kept for larvae collected, percent pupariation, and death of adult flies on each day, and tabulated for analysis.

### Wasp parasitism and survival assay

Wasp infestation was performed following standardized protocol as described in Rodrigues et al. (2021a). Briefly, second-instar larvae (about 48-52 h, AEL) were exposed to the parasitoid wasp species *Leptopilina boulardi* (provided by Tina Mukherjee, inStem) at a ratio of one female wasp per eight *Drosophila* larvae, in vials, for 3 h. *Drosophila* larvae were then allowed to develop for 72 h and dissected to check infestation. As *domeGal4 UAS-2xEGFP* driver was used, GFP positive larvae were screened for both control and knockdown crosses. *Drosophila* larvae that contained at least two wasp larvae in the hemocoel were analyzed for the presence of lamellocytes by staining for Phalloidin and L1, as described.

For survival assay, 10 female L. boulardi from a running culture were introduced into vials with 40-50 second-instar larvae. Wasps were removed after 3 h. Fly larvae were allowed to develop for 3 days to the late third-instar stage, and then GFP+ve larvae were collected for further observation. Number of eclosing adult flies was recorded for calculating percentage of survival.

The genotypes used were as follows:

With *domeGal4 2xEGFP* driver- control: *domeGal4, UAS-2xEGFP>/Fm7a; +; +,* Vps25 KD: *domeGal4, UAS-2xEGFP/+; UAS-Vps25 RNAi/+; +,* Vps36 KD: *domeGal4, UAS-2xEGFP/+; UAS Vps36 RNAi/+; +*.

With elavGal80;;domeMESOGFP> driver- control: elavGal80*; +;* domeMESOGFP>/+*,* Vps25 KD: elavGal80*; UAS-Vps25 RNAi*/+*;* domeMESOGFP>/+*,* Vps36 KD: elavGal80*; UAS Vps36 RNAi*/+*;* domeMESOGFP>/+*,* Vps32 KD: elavGal80; *UAS-Vps32 RNAi/+;* domeMESOGFP>/+.

### Proximity ligation assay

PLA was performed using the manufacturer's recommended protocol, as described before (Khadilkar et al., 2014). Rabbit plus and mouse minus probes were used along with orange DuoLink PLA kit (Merck, USA). Rabbit anti-Vps28 (Helmut Kramer, UT Southwestern Medical Center) or anti-Vps32 (Fen B. Gao, University of Massachusetts Medical School) and mouse anti-NICD (DSHB, USA) antibodies were used.

### Quantification

Fluorescence intensity across lymph gland lobes was quantified using Fiji (ImageJ) to estimate any difference in protein expression. Number of ubiquitin aggregates in each lobe was quantified manually and then normalized over DAPI-positive nuclei count to obtain the fraction of cells accumulating ubiquitinated cargoes in each lobe. Blood cell differentiation was quantified using Imaris (Bitplane) as described in Ray et al. (2021). Briefly, the number of spots (DAPI positive nuclei with >2 µm diameter) close to the reconstructed dome>2xEGFP (for prohemocytes) or P1 (for plasmatocytes) surface, by a set threshold distance (1 µm for prohemocytes and 2 µm for plasmatocytes), was quantified and divided by total number of nuclei. The number of crystal cells in each lobe was quantified manually and its fraction was calculated in each lobe by dividing with the number of nuclei. Lamellocytes were identified based on large or elongated morphology as revealed by Phalloidin staining. Percentage of larvae showing lamellocyte differentiation was quantified and represented.

The number of cells with high NRE-GFP expression was quantified manually in each lobe. For quantifying NICD accumulation, cells with intracellular NICD accumulation (not commonly seen in control lymph glands) were counted in each lobe. To determine the fraction of cells undergoing Notch activation or crystal cell differentiation, the number of cells expressing high level of NRE-GFP or ProPO was normalized over DAPI positive nuclei count. The number of PLA dots (see Supplementary material) per 100 µm^2^ area of the progenitor subsets across primary, secondary and tertiary lobes of the lymph gland was manually counted. Mitotic potential was determined by estimating the number of nuclei with high H3P expression (above a set threshold value). Lymph glands were binned in three categories based on the lobe margin: regular, irregular/disintegrated, tumorous bulge and percentage of lymph gland under each category was estimated. All images within a given figure panel were adjusted equally for brightness and contrast using Adobe Photoshop CS5 extended. GraphPad Prism 8 and MS Excel were used to prepare the graphs. Autodesk Sketchbook version 5.2.2 was used to prepare schematics in Fig. 5C.

### Statistical analyses

Each larva was considered as a biological replicate. Data from each lymph gland lobe were individually considered for quantitation in all graphs. One-way ANOVA was performed for statistical analysis of data. For datasets with unequal variance across groups, non-parametric tests such as Kruskal–Wallis test were performed.

## Supplementary Material

10.1242/biolopen.060412_sup1 Supplementary information

Table S1.

## References

[BIO060412C1] Adoro, S., Park, K. H., Bettigole, S. E., Lis, R., Shin, H. R., Seo, H., Kim, J. H., Knobeloch, K. P., Shim, J. H. and Glimcher, L. H. (2017). Post-translational control of T cell development by the ESCRT protein CHMP5. *Nat. Immunol.* 18, 780-790. 10.1038/ni.3764 28553951

[BIO060412C2] Alfred, V. and Vaccari, T. (2016). When membranes need an ESCRT: endosomal sorting and membrane remodelling in health and disease. *Swiss Med. Wkly.* 146, w14347. 10.4414/smw.2016.14347 27631343

[BIO060412C3] Avet-Rochex, A., Boyer, K., Polesello, C., Gobert, V., Osman, D., Roch, F., Auge, B., Zanet, J., Haenlin, M. and Waltzer, L. (2010). An in vivo RNA interference screen identifies gene networks controlling Drosophila melanogaster blood cell homeostasis. *BMC Dev. Biol.* 10, 65. 10.1186/1471-213X-10-65 20540764 PMC2891661

[BIO060412C4] Baumeister, J., Maie, T., Chatain, N., Gan, L., Weinbergerova, B., De Toledo, M. A. S., Eschweiler, J., Maurer, A., Mayer, J., Kubesova, B. et al. (2021). Early and late stage MPN patients show distinct gene expression profiles in CD34(+) cells. *Ann. Hematol.* 100, 2943-2956. 10.1007/s00277-021-04615-8 34390367 PMC8592960

[BIO060412C5] Benmimoun, B., Polesello, C., Haenlin, M. and Waltzer, L. (2015). The EBF transcription factor Collier directly promotes Drosophila blood cell progenitor maintenance independently of the niche. *Proc. Natl. Acad. Sci. USA* 112, 9052-9057. 10.1073/pnas.1423967112 26150488 PMC4517242

[BIO060412C6] Bissig, C. and Gruenberg, J. (2014). ALIX and the multivesicular endosome: ALIX in Wonderland. *Trends Cell Biol.* 24, 19-25. 10.1016/j.tcb.2013.10.009 24287454

[BIO060412C7] Blanco-Obregon, D., Katz, M. J., Durrieu, L., Gandara, L. and Wappner, P. (2020). Context-specific functions of Notch in Drosophila blood cell progenitors. *Dev. Biol.* 462, 101-115. 10.1016/j.ydbio.2020.03.018 32243888

[BIO060412C8] Di Fiore, P. P. and Von Zastrow, M. (2014). Endocytosis, signaling, and beyond. *Cold Spring Harb. Perspect. Biol.* 6, a016865. 10.1101/cshperspect.a016865 25085911 PMC4107983

[BIO060412C9] Diederich, R. J., Matsuno, K., Hing, H. and Artavanis-Tsakonas, S. (1994). Cytosolic interaction between deltex and Notch ankyrin repeats implicates deltex in the Notch signaling pathway. *Development* 120, 473-481. 8162848 10.1242/dev.120.3.473

[BIO060412C10] Duvic, B., Hoffmann, J. A., Meister, M. and Royet, J. (2002). Notch signaling controls lineage specification during Drosophila larval hematopoiesis. *Curr. Biol.* 12, 1923-1927. 10.1016/s0960-9822(02)01297-6 12445385

[BIO060412C11] Gorombei, P., Guidez, F., Ganesan, S., Chiquet, M., Pellagatti, A., Goursaud, L., Tekin, N., Beurlet, S., Patel, S., Guerenne, L. et al. (2021). BCL-2 Inhibitor ABT-737 effectively targets leukemia-initiating cells with differential regulation of relevant genes leading to extended survival in a NRAS/BCL-2 mouse model of high risk-myelodysplastic syndrome. *Int. J. Mol. Sci.* 22, 10658. 10.3390/ijms221910658 34638998 PMC8508829

[BIO060412C12] Hariharan, I. K. and Bilder, D. (2006). Regulation of imaginal disc growth by tumor-suppressor genes in Drosophila. *Annu. Rev. Genet.* 40, 335-361. 10.1146/annurev.genet.39.073003.100738 16872256

[BIO060412C13] Herz, H. M., Chen, Z., Scherr, H., Lackey, M., Bolduc, C. and Bergmann, A. (2006). vps25 mosaics display non-autonomous cell survival and overgrowth, and autonomous apoptosis. *Development* 133, 1871-1880. 16611691 10.1242/dev.02356 PMC2519036

[BIO060412C14] Herz, H. M., Woodfield, S. E., Chen, Z., Bolduc, C. and Bergmann, A. (2009). Common and distinct genetic properties of ESCRT-II components in Drosophila. *PLoS One* 4, e4165. 10.1371/journal.pone.0004165 19132102 PMC2613530

[BIO060412C15] Ho, K. Y. L., Carr, R. L., Dvoskin, A. D. and Tanentzapf, G. (2023). Kinetics of blood cell differentiation during hematopoiesis revealed by quantitative long-term live imaging. *Elife* 12, e84085. 10.7554/eLife.84085 37000163 PMC10065797

[BIO060412C16] Hori, K., Sen, A., Kirchhausen, T. and Artavanis-Tsakonas, S. (2011). Synergy between the ESCRT-III complex and Deltex defines a ligand-independent Notch signal. *J. Cell Biol.* 195, 1005-1015. 10.1083/jcb.201104146 22162134 PMC3241730

[BIO060412C17] Hori, K., Sen, A., Kirchhausen, T. and Artavanis-Tsakonas, S. (2012). Regulation of ligand-independent Notch signal through intracellular trafficking. *Commun. Integr. Biol.* 5, 374-376. 10.4161/cib.19995 23060962 PMC3460843

[BIO060412C18] Horner, D. S., Pasini, M. E., Beltrame, M., Mastrodonato, V., Morelli, E. and Vaccari, T. (2018). ESCRT genes and regulation of developmental signaling. *Semin. Cell Dev. Biol.* 74, 29-39. 10.1016/j.semcdb.2017.08.038 28847745

[BIO060412C19] Itoh, M., Kim, C. H., Palardy, G., Oda, T., Jiang, Y. J., Maust, D., Yeo, S. Y., Lorick, K., Wright, G. J., Ariza-Mcnaughton, L. et al. (2003). Mind bomb is a ubiquitin ligase that is essential for efficient activation of Notch signaling by Delta. *Dev. Cell* 4, 67-82. 10.1016/s1534-5807(02)00409-4 12530964

[BIO060412C20] Khadilkar, R. J., Rodrigues, D., Mote, R. D., Sinha, A. R., Kulkarni, V., Magadi, S. S. and Inamdar, M. S. (2014). ARF1-GTP regulates Asrij to provide endocytic control of Drosophila blood cell homeostasis. *Proc. Natl. Acad. Sci. USA* 111, 4898-4903. 10.1073/pnas.1303559111 24707047 PMC3977295

[BIO060412C21] Kulkarni, V., Khadilkar, R. J., Magadi, S. S. and Inamdar, M. S. (2011). Asrij maintains the stem cell niche and controls differentiation during Drosophila lymph gland hematopoiesis. *PLoS One* 6, e27667. 10.1371/journal.pone.0027667 22110713 PMC3215734

[BIO060412C22] Lai, E. C., Deblandre, G. A., Kintner, C. and Rubin, G. M. (2001). Drosophila neuralized is a ubiquitin ligase that promotes the internalization and degradation of delta. *Dev. Cell* 1, 783-794. 10.1016/s1534-5807(01)00092-2 11740940

[BIO060412C23] Lebestky, T., Jung, S. H. and Banerjee, U. (2003). A Serrate-expressing signaling center controls Drosophila hematopoiesis. *Genes Dev.* 17, 348-353. 10.1101/gad.1052803 12569125 PMC195988

[BIO060412C24] Liu, Y., Mei, Y., Han, X., Korobova, F. V., Prado, M. A., Yang, J., Peng, Z., Paulo, J. A., Gygi, S. P., Finley, D. et al. (2021). Membrane skeleton modulates erythroid proteome remodeling and organelle clearance. *Blood* 137, 398-409. 10.1182/blood.2020006673 33036023 PMC7819763

[BIO060412C25] Mandal, L., Martinez-Agosto, J. A., Evans, C. J., Hartenstein, V. and Banerjee, U. (2007). A Hedgehog- and Antennapedia-dependent niche maintains Drosophila haematopoietic precursors. *Nature* 446, 320-324. 10.1038/nature05585 17361183 PMC2807630

[BIO060412C26] Matsuno, K., Diederich, R. J., Go, M. J., Blaumueller, C. M. and Artavanis-Tsakonas, S. (1995). Deltex acts as a positive regulator of Notch signaling through interactions with the Notch ankyrin repeats. *Development* 121, 2633-2644. 7671825 10.1242/dev.121.8.2633

[BIO060412C27] Mattissek, C. and Teis, D. (2014). The role of the endosomal sorting complexes required for transport (ESCRT) in tumorigenesis. *Mol. Membr. Biol.* 31, 111-119. 10.3109/09687688.2014.894210 24641493 PMC4059258

[BIO060412C28] Moretti, J., Chastagner, P., Gastaldello, S., Heuss, S. F., Dirac, A. M., Bernards, R., Masucci, M. G., Israel, A. and Brou, C. (2010). The translation initiation factor 3f (eIF3f) exhibits a deubiquitinase activity regulating Notch activation. *PLoS Biol.* 8, e1000545. 10.1371/journal.pbio.1000545 21124883 PMC2990700

[BIO060412C29] Morin-Poulard, I., Vincent, A. and Crozatier, M. (2013). The Drosophila JAK-STAT pathway in blood cell formation and immunity. *JAKSTAT* 2, e25700. 10.4161/jkst.25700 24069567 PMC3772119

[BIO060412C30] Olmos, Y., Hodgson, L., Mantell, J., Verkade, P. and Carlton, J. G. (2015). ESCRT-III controls nuclear envelope reformation. *Nature* 522, 236-239. 10.1038/nature14503 26040713 PMC4471131

[BIO060412C31] Pashkova, N., Gakhar, L., Winistorfer, S. C., Sunshine, A. B., Rich, M., Dunham, M. J., Yu, L. and Piper, R. C. (2013). The yeast Alix homolog Bro1 functions as a ubiquitin receptor for protein sorting into multivesicular endosomes. *Dev. Cell* 25, 520-533. 10.1016/j.devcel.2013.04.007 23726974 PMC3755756

[BIO060412C32] Pellagatti, A., Cazzola, M., Giagounidis, A., Perry, J., Malcovati, L., Della Porta, M. G., Jadersten, M., Killick, S., Verma, A., Norbury, C. J. et al. (2010). Deregulated gene expression pathways in myelodysplastic syndrome hematopoietic stem cells. *Leukemia* 24, 756-764. 10.1038/leu.2010.31 20220779

[BIO060412C33] Radulovic, M., Schink, K. O., Wenzel, E. M., Nahse, V., Bongiovanni, A., Lafont, F. and Stenmark, H. (2018). ESCRT-mediated lysosome repair precedes lysophagy and promotes cell survival. *EMBO J.* 37, e99753. 30314966 10.15252/embj.201899753 PMC6213280

[BIO060412C34] Ray, A. (2021). Elucidating the role of mitochondrial dynamics and endosomal protein sorting in drosophila blood cell homeostasis. *PhD Thesis*, Jawaharlal Nehru Centre For Advanced Scientific Research.

[BIO060412C35] Ray, A., Kamat, K. and Inamdar, M. S. (2021). A conserved role for Asrij/OCIAD1 in progenitor differentiation and lineage specification through functional interaction with the regulators of mitochondrial dynamics. *Front. Cell Dev. Biol.* 9, 643444. 10.3389/fcell.2021.643444 34295888 PMC8290362

[BIO060412C36] Reimels, T. A. and Pfleger, C. M. (2015). Drosophila Rabex-5 restricts Notch activity in hematopoietic cells and maintains hematopoietic homeostasis. *J. Cell Sci.* 128, 4512-4525. 10.1242/jcs.174433 26567216 PMC4696494

[BIO060412C37] Rodrigues, D., Renaud, Y., Vijayraghavan, K., Waltzer, L. and Inamdar, M. S. (2021a). Differential activation of JAK-STAT signaling reveals functional compartmentalization in Drosophila blood progenitors. *Elife* 10, e61409. 10.7554/eLife.61409 33594977 PMC7920551

[BIO060412C38] Rodrigues, D., Vijayraghavan, K., Waltzer, L. and Inamdar, M. S. (2021b). Intact in situ preparation of the drosophila melanogaster lymph gland for a comprehensive analysis of larval hematopoiesis. *Bio-protocol* 11, e4204. 10.21769/BioProtoc.4204 34859119 PMC8595419

[BIO060412C39] Shravage, B. V., Hill, J. H., Powers, C. M., Wu, L. and Baehrecke, E. H. (2013). Atg6 is required for multiple vesicle trafficking pathways and hematopoiesis in Drosophila. *Development* 140, 1321-1329. 10.1242/dev.089490 23406899 PMC3585664

[BIO060412C40] Stempels, F. C., Jiang, M., Warner, H. M., Moser, M. L., Janssens, M. H., Maassen, S., Nelen, I. H., De Boer, R., Jiemy, W. F., Knight, D. et al. (2023). Giant worm-shaped ESCRT scaffolds surround actin-independent integrin clusters. *J. Cell Biol.* 222, e202205130. 10.1083/jcb.202205130 37200023 PMC10200693

[BIO060412C41] Stephenson, H. N., Streeck, R., Grublinger, F., Goosmann, C. and Herzig, A. (2022). Hemocytes are essential for Drosophila melanogaster post-embryonic development, independent of control of the microbiota. *Development* 149, dev200286. 10.1242/dev.200286 36093870 PMC9641648

[BIO060412C42] Szymanska, E., Budick-Harmelin, N. and Miaczynska, M. (2018). Endosomal "sort" of signaling control: The role of ESCRT machinery in regulation of receptor-mediated signaling pathways. *Semin. Cell Dev. Biol.* 74, 11-20. 10.1016/j.semcdb.2017.08.012 28797837

[BIO060412C43] Tabernero, L. and Woodman, P. (2018). Dissecting the role of His domain protein tyrosine phosphatase/PTPN23 and ESCRTs in sorting activated epidermal growth factor receptor to the multivesicular body. *Biochem. Soc. Trans.* 46, 1037-1046. 10.1042/BST20170443 30190330 PMC6195633

[BIO060412C44] Tognon, E., Wollscheid, N., Cortese, K., Tacchetti, C. and Vaccari, T. (2014). ESCRT-0 is not required for ectopic Notch activation and tumor suppression in Drosophila. *PLoS One* 9, e93987. 10.1371/journal.pone.0093987 24718108 PMC3981749

[BIO060412C45] Tsunematsu, T., Yamauchi, E., Shibata, H., Maki, M., Ohta, T. and Konishi, H. (2010). Distinct functions of human MVB12A and MVB12B in the ESCRT-I dependent on their posttranslational modifications. *Biochem. Biophys. Res. Commun.* 399, 232-237. 10.1016/j.bbrc.2010.07.060 20654576

[BIO060412C46] Vaccari, T. and Bilder, D. (2005). The Drosophila tumor suppressor vps25 prevents nonautonomous overproliferation by regulating notch trafficking. *Dev. Cell* 9, 687-698. 10.1016/j.devcel.2005.09.019 16256743

[BIO060412C47] Vaccari, T., Rusten, T. E., Menut, L., Nezis, I. P., Brech, A., Stenmark, H. and Bilder, D. (2009). Comparative analysis of ESCRT-I, ESCRT-II and ESCRT-III function in Drosophila by efficient isolation of ESCRT mutants. *J. Cell Sci.* 122, 2413-2423. 10.1242/jcs.046391 19571114 PMC2704878

[BIO060412C48] Vietri, M., Radulovic, M. and Stenmark, H. (2019). The many functions of ESCRTs. *Nat. Rev. Mol. Cell Biol* 21, 25-42. 10.1038/s41580-019-0177-4 31705132

[BIO060412C49] Wang, Y., Chen, Z. and Bergmann, A. (2010). Regulation of EGFR and Notch signaling by distinct isoforms of D-cbl during Drosophila development. *Dev. Biol.* 342, 1-10. 10.1016/j.ydbio.2010.03.005 20302857 PMC2866751

[BIO060412C50] Wang, J., Fang, N., Xiong, J., Du, Y., Cao, Y. and Ji, W. K. (2021). An ESCRT-dependent step in fatty acid transfer from lipid droplets to mitochondria through VPS13D-TSG101 interactions. *Nat. Commun.* 12, 1252. 10.1038/s41467-021-21525-5 33623047 PMC7902631

[BIO060412C51] Xia, P., Wang, S., Huang, G., Zhu, P., Li, M., Ye, B., Du, Y. and Fan, Z. (2014). WASH is required for the differentiation commitment of hematopoietic stem cells in a c-Myc-dependent manner. *J. Exp. Med.* 211, 2119-2134. 10.1084/jem.20140169 25225459 PMC4172220

[BIO060412C52] Yu, S., Luo, F. and Jin, L. H. (2021). Rab5 and Rab11 maintain hematopoietic homeostasis by restricting multiple signaling pathways in Drosophila. *Elife* 10, e60870. 10.7554/eLife.60870 33560224 PMC7891935

