# Supplementary material for: The Endosomal Sorting Complex, ESCRT, has diverse roles in blood progenitor maintenance, lineage choice and immune response
Source: Biol Open. 2024 Jun 18;13(6):bio060412. doi: 10.1242/bio.060412 (PMC11212638; doi:10.1242/bio.060412)
Supplement: Supplementary information [file biolopen-13-060412-s1.pdf]

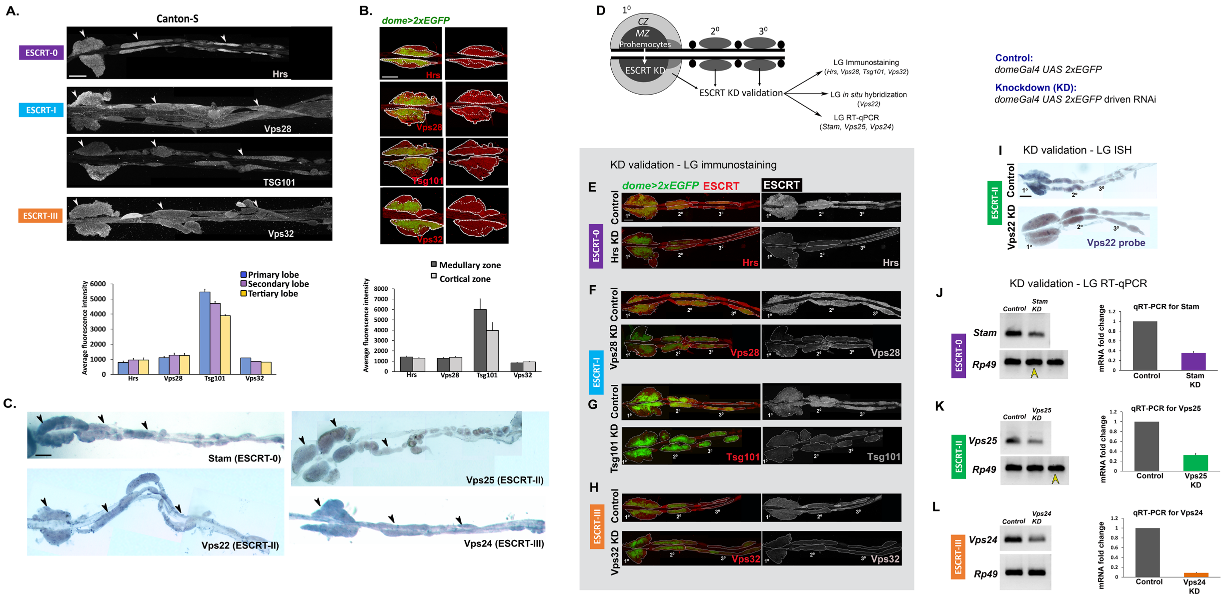

Supplementary Figure S1

Fig. S1. ESCRT is uniformly expressed in the lymph gland and downregulated after RNAi.

(A) Immunofluorescence microscopy of whole mount Canton-S lymph glands showing expression of ESCRT components Hrs (ESCRT-0), Vps28 and Tsg101 (ESCRT-I) and Vps32 (ESCRT-III) across different lobes of the lymph gland. Arrowheads mark the primary, secondary and tertiary lobes. Bar diagram shows quantification of mean fluorescence intensity for immunostaining of ESCRT components across three lobes. **(B)** dome>2xEGFP+ve region marks prohemocytes in the medullary zone demarcated by dotted line in the primary lobe. Immunostaining is shown for Hrs, Vps28, Tsg101 and Vps32. Bar diagrams show quantification and comparison of mean fluorescence intensity of a given component in dome>2xEGFP+ve medullary zone and dome>2xEGFP-ve cortical zone. **(C)** RNA *in situ* hybridisation shows expression of ESCRT components Stam (ESCRT-0), Vps22 and Vps25 (ESCRT-II) and Vps24 (ESCRT-III) at transcript level across different lobes. Arrowheads mark the different lobes. Scale bar: 100 µm. N>5 larvae with each individual lobes analysed. Error bars in the graph represent SEM. One-way ANOVA was performed to determine the statistical significance. **(D-H)** Validation of *domeGal4*-driven knockdown of ESCRT components was performed using immunofluorescence microscopy, *in situ* hybridisation and RT-qPCR (D). Immunostaining using respective antibodies shows knockdown of ESCRT component Hrs (ESCRT-0) (E), Vps28 (F) and Tsg101 (ESCRT-I) (G) and Vps32 (ESCRT-III) (H). **(I)** RNA *in situ* hybridisation shows knockdown of Vps22 (ESCRT-II). **(J-L)** RT-qPCR from lymph gland validates knockdown of Stam (ESCRT-0), Vps25 (ESCRT-II) and Vps24 (ESCRT-III). Arrowheads mark Rp49 PCR band for corresponding sample. Scale bar: 100 µm. N>5 larvae for IF and N=10 larvae for ISH-based validation. RT-qPCR was performed in triplicates using RNA isolated from 100 lymph glands for each genotype. Error bars represent SEM.

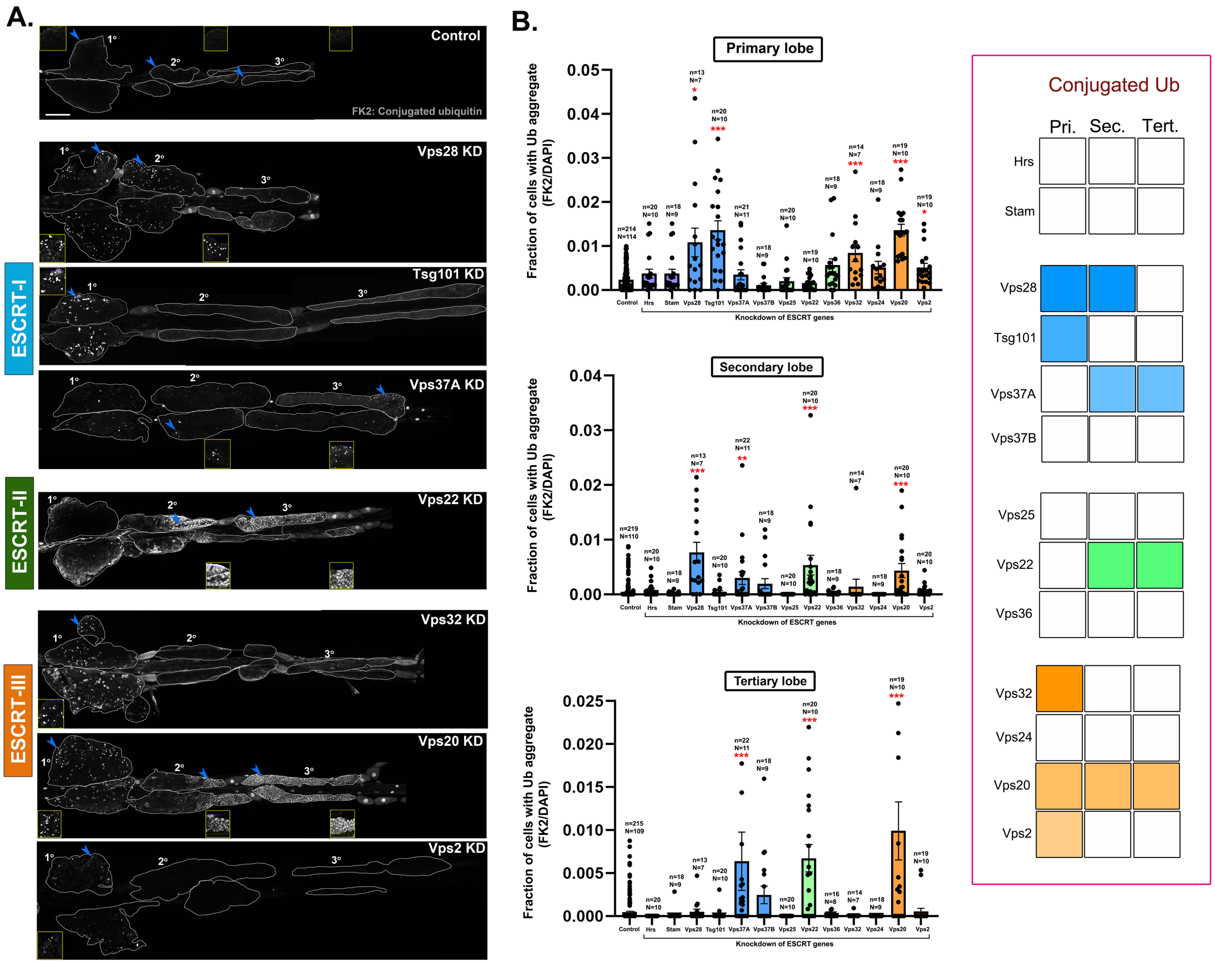

Supplementary Figure S2

Fig. S2. ESCRT components regulate ubiquitinated cargo sorting in the lymph gland.

(A) Whole-mount larval lymph gland showing accumulation of conjugated ubiquitin (FK2) in the lymph gland upon progenitor-specific (*domeGal4 UAS 2xEGFP* driven) knockdown of 7 *Drosophila* ESCRT components indicated (Vps28, Tsg101, Vps37A, Vps22, Vps32, Vps20, Vps2). Ubiquitin staining is shown in gray scale. Accumulation of ubiquitin aggregates is marked by arrowhead and magnified in insets. Scale bar: 100 µm. **(B)** Bar diagrams show quantification of the fraction of cells accumulating ubiquitin aggregates in primary, secondary and tertiary lobes upon knockdown of all 13 core ESCRT components. n indicates the number of individual lobes analysed and N indicates the number of larvae analysed. Error bars represent SEM. Kruskal Wallis test was performed to determine the statistical significance. *P<0.05, P**<0.01, ***P<0.001. Summary chart indicating presence (colored box) or absence (white box) of ubiquitin accumulation in the primary, secondary and tertiary lobes upon depletion of the respective ESCRT component (left).

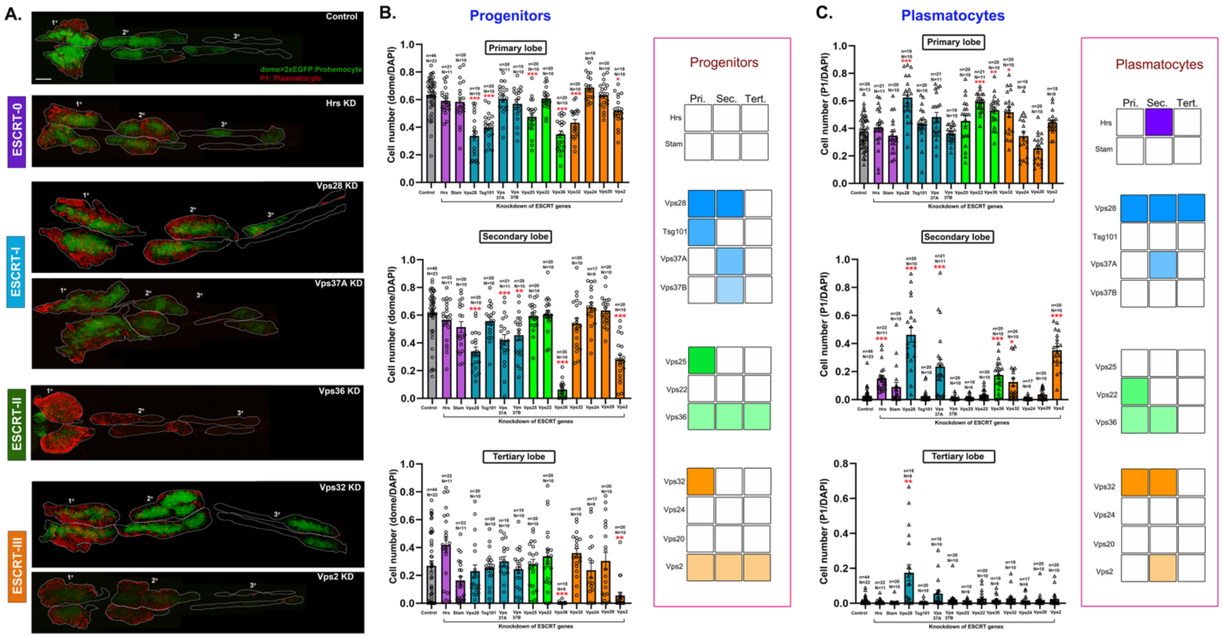

Supplementary Figure S3

Fig. S3. ESCRT components regulate progenitor maintenance and plasmatocyte differentiation in the lymph gland.

(A) Whole-mount larval lymph gland showing change in the fraction of dome>2xEGFP+ve progenitors (green) or P1+ve plasmatocytes (red) in the lymph gland upon progenitor-specific knockdown of 6 ESCRT components (Hrs, Vps28, Vps37A, Vps36, Vps32, Vps2). Scale bar: 100 µm. **(B-C)** Bar diagrams show quantification of the fraction of progenitors (B) and plasmatocytes (C) in primary, secondary and tertiary lobes upon knockdown of all 13 core ESCRT components. n indicates the number of individual lobes analysed and N indicates the number of larvae analysed. Error bars represent SEM. Kruskal Wallis test was performed to determine the statistical significance. *P<0.05, **P<0.01, ***P<0.001. Summary chart indicating presence (colored box) or absence (white box) of phenotypes of progenitor loss (B) or increased plasmatocytes differentiation (C) in the primary, secondary and tertiary lobes upon depletion of the respective ESCRT component (left).

Biology Open • Supplementary information

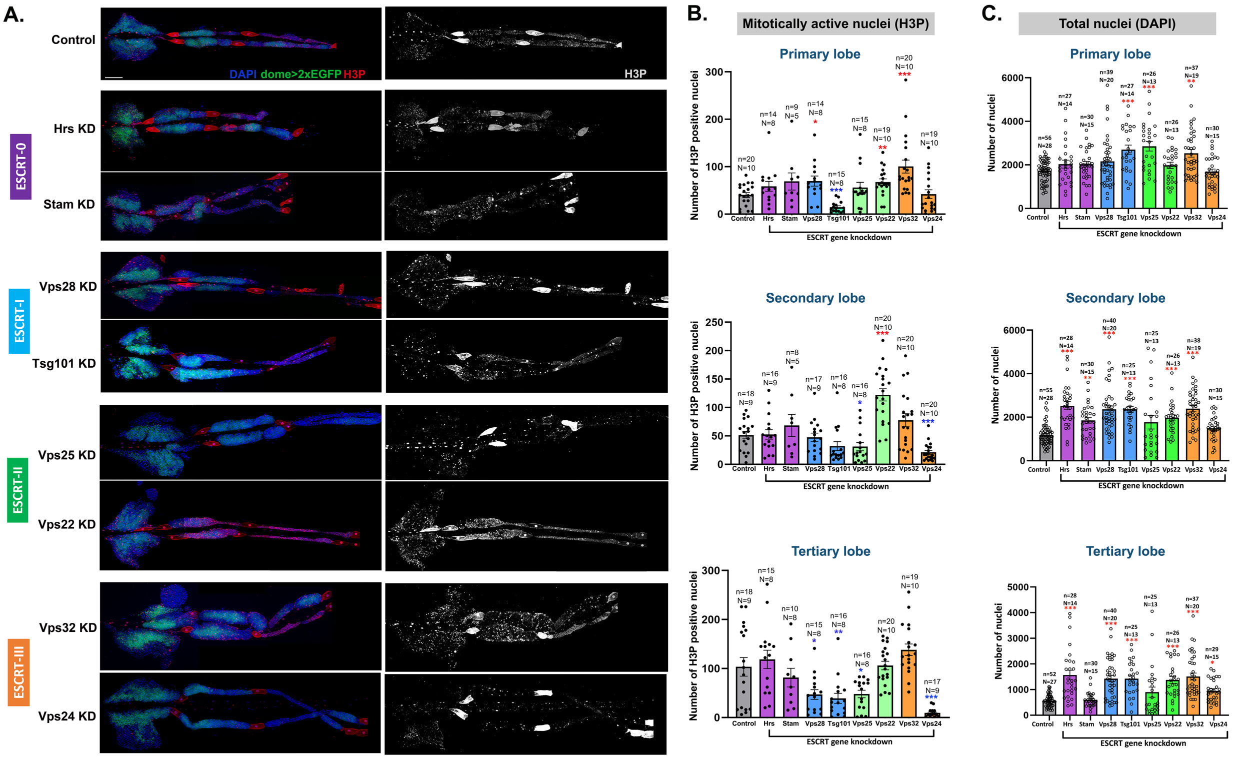

Supplementary Figure S4

Fig. S4. ESCRT components differentially regulate mitotic potential and tissue size across the lymph gland.

(A) Whole-mount larval lymph gland showing immunostaining for phosphorylated Histone H3 to mark mitotically active nuclei (red in the left image panel and grayscale in the right image panel) upon progenitor-specific knockdown of 8 ESCRT components [Hrs, Stam (ESCRT-0); Vps28, Tsg101 (ESCRT-I); Vps25, Vps22 (ESCRT-II); Vps32, Vps24 (ESCRT-III)]. Scale bar: 100 µm. **(B)** Bar diagrams show quantification of the number of H3P positive (high H3P) nuclei in primary, secondary and tertiary lobes of the same genotypes. **(C)** The total number of nuclei in each lobe has also been quantified for each lobe in the same genotypes. n indicates the number of individual lobes analysed and N indicates the number of larvae analysed. Error bars represent SEM. One-way ANOVA was performed to determine the statistical significance. *P<0.05, **P<0.01, ***P<0.001.

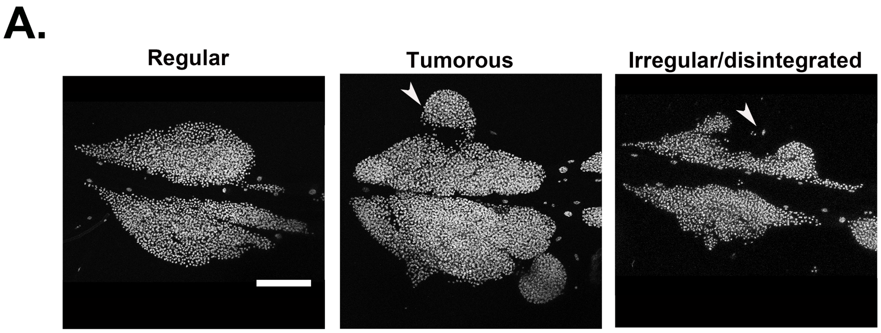

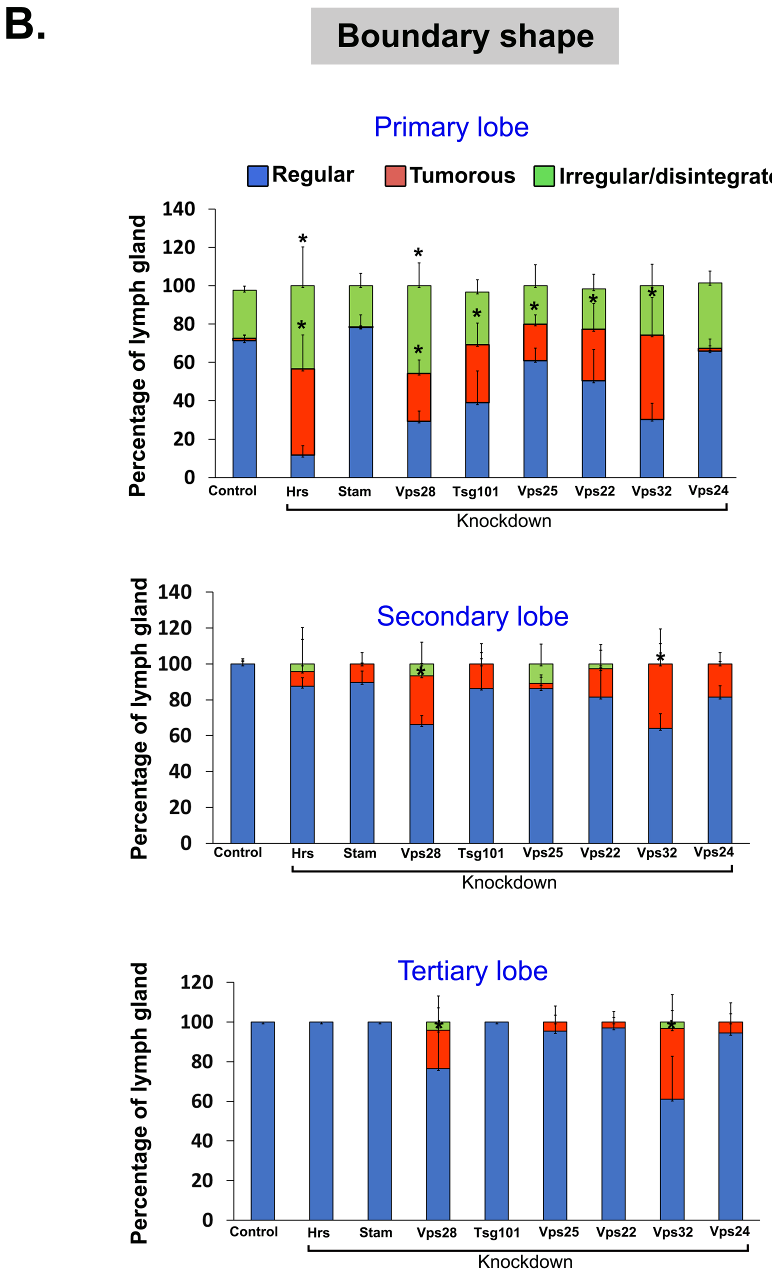

Supplementary Figure S5

Biology Open • Supplementary information

Fig. S5. ESCRT components affect the morphology of the lymph gland lobes.

(A) Representative primary lobe images of the lymph gland showing regular boundary, irregular/ disintegrated boundary and tumorous bulge. Scale bar: 100 µm. **(B)** Quantification of the percentage of larvae showing aforementioned morphology of the primary, secondary and tertiary lobes for knockdown of 8 ESCRT genes [Hrs, Stam (ESCRT-0); Vps28, Tsg101 (ESCRT-I); Vps25, Vps22 (ESCRT-II); Vps32, Vps24 (ESCRT-III)]. N>30 for each genotype. Error bars represent SEM. One-way ANOVA was performed to determine the statistical significance. *P<0.05.

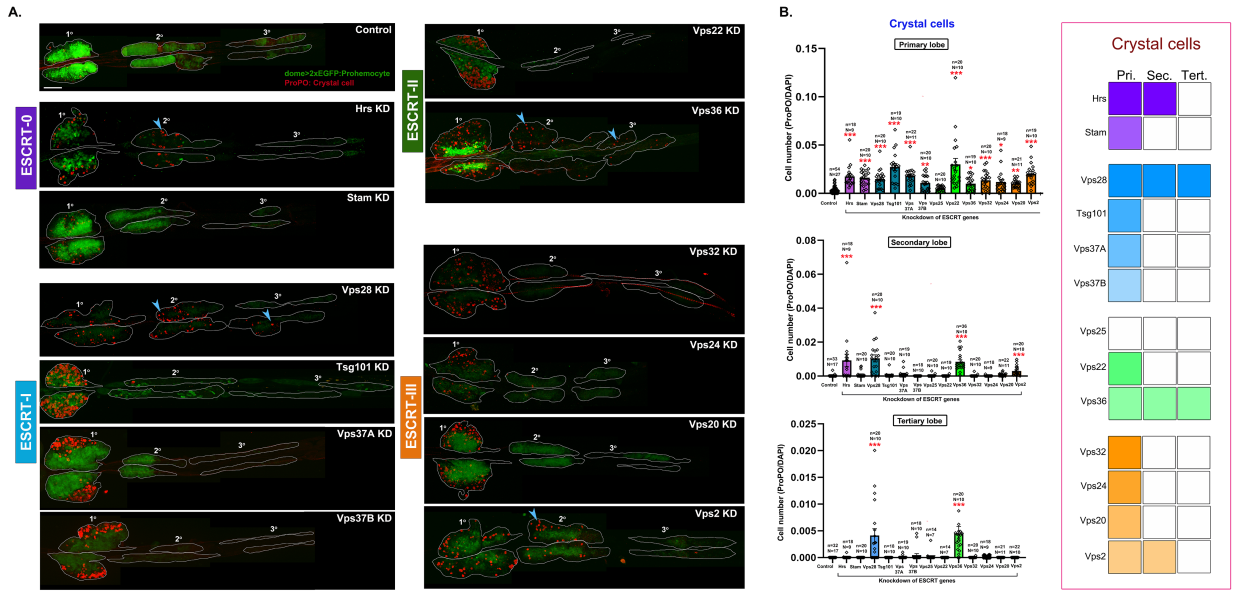

Supplementary Figure S6

Fig. S6. ESCRT components differentially regulate crystal cell differentiation of lymph gland progenitors.

(A) Whole-mount larval lymph gland showing differentiation of ProPO+ve crystal cells (red) in the lymph gland upon progenitor-specific knockdown of 12 core ESCRT components. Dome>2xEGFP (green) marks the progenitors across different lobes. Arrowheads mark presence of crystal cells in posterior lobes. Scale bar: 100 µm. **(B)** Bar diagram shows quantification of the fraction of crystal cells in primary, secondary and tertiary lobes upon knockdown of all 13 core ESCRT components. n indicates the number of individual lobes analysed and N indicates the number of larvae analysed. Error bars represent SEM. Kruskal Wallis test was performed to determine the statistical significance. *P<0.05, **P<0.01, ***P<0.001. Summary chart indicating presence (colored box) or absence (white box) of phenotypes of increased crystal cell differentiation in the primary, secondary and tertiary lobes upon depletion of the respective ESCRT component (left). Also see Fig. 1.

Biology Open • Supplementary information

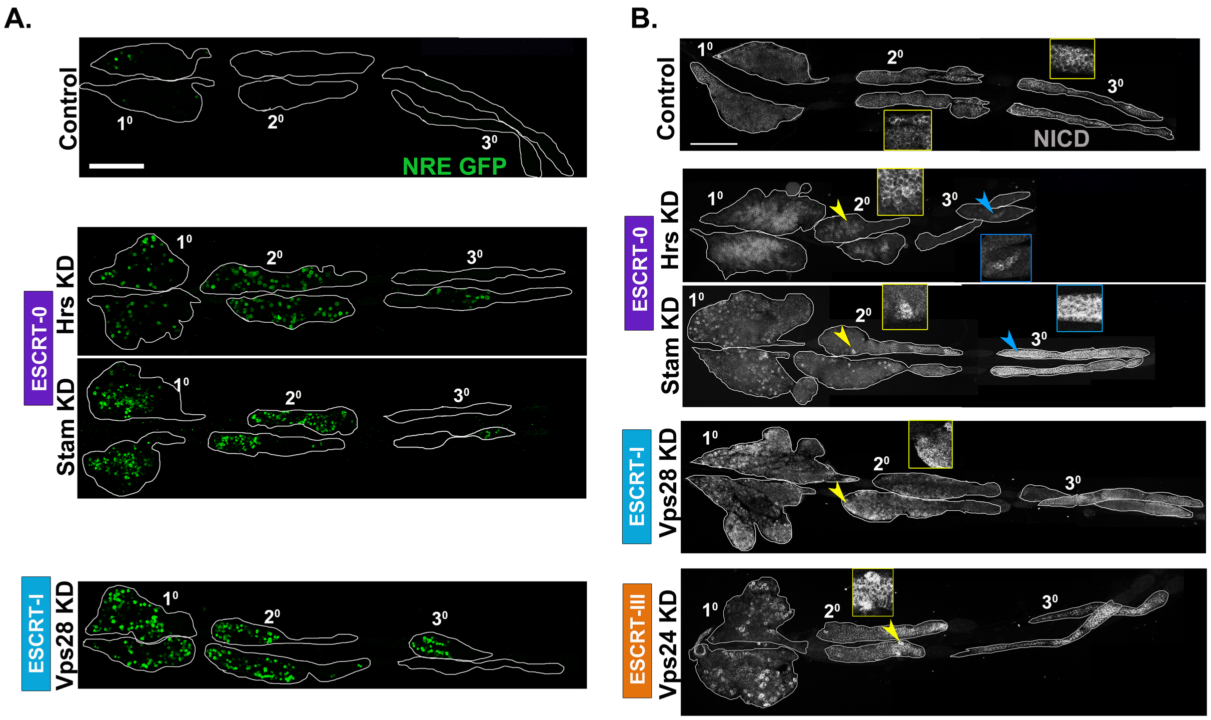

Supplementary Figure S7

Fig. S7. ESCRT regulates Notch activation and NICD trafficking in the lymph gland. (related to Figure 2)

Whole-mount larval lymph gland showing NRE-GFP staining to mark Notch activation across all lobes upon progenitor-specific knockdown of ESCRT components Hrs, Stam (ESCRT-0) and Vps28 (ESCRT-I). Lymph glands in the adjacent panel show NICD staining across the lymph gland upon progenitor-specific knockdown of ESCRT components Hrs, Stam (ESCRT-0) and Vps28 (ESCRT-I). NICD accumulation in secondary (yellow arrowhead) and tertiary lobes (blue arrowhead) are shown in the insets. Scale bar: 100 µm.

Biology Open • Supplementary information

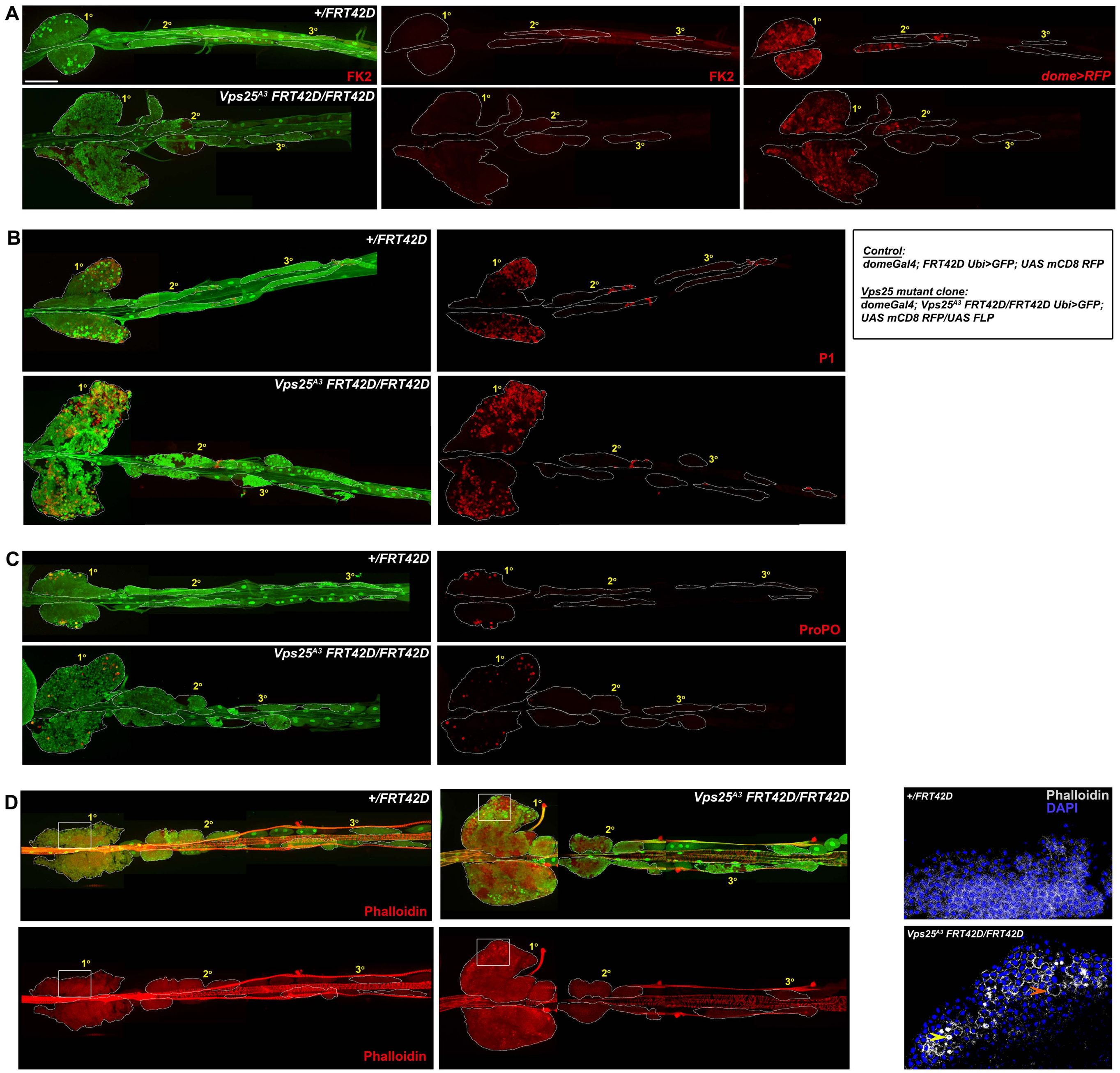

Supplementary Figure S8

Fig. S8. Vps25 mutation does not affect ubiquitination and blood cell differentiation in the lymph gland. (related to Figure 1, S3, S5, S6 and S9).

(A) Whole mount lymph gland showing staining for conjugated ubiquitin in control (*domeGal4; FRT42D Ubi>GFP; UAS mCD8 RFP*) and Vps25 mutant clone (*domeGal4/+; Vps25^{A3}FRT42D/FRT42D Ubi>GFP; UAS mCD8 RFP/UAS FLP)* lymph gland. GFP expression marks the wild type twin-spot while GFP negative region marks the homozygous mutant clone. dome>RFP marks the progenitor in the same genotype, **(B)** P1

Biology Open • Supplementary information

marks plasmatocytes, and **(C)** ProPO marks crystal cells. **(D)** Phalloidin staining was used to visualize lamellocytes based on their elongated morphology. Bottom-most panel shows enlarged view of the boxed region from control and mutant lymph glands. Phalloidin staining in shown in grayscale. DAPI marks the nuclei. The orange arrowhead marks a big binucleate cell while the yellow arrowhead marks a very small cell with high F-actin expression. Scale bar: 100 µm.

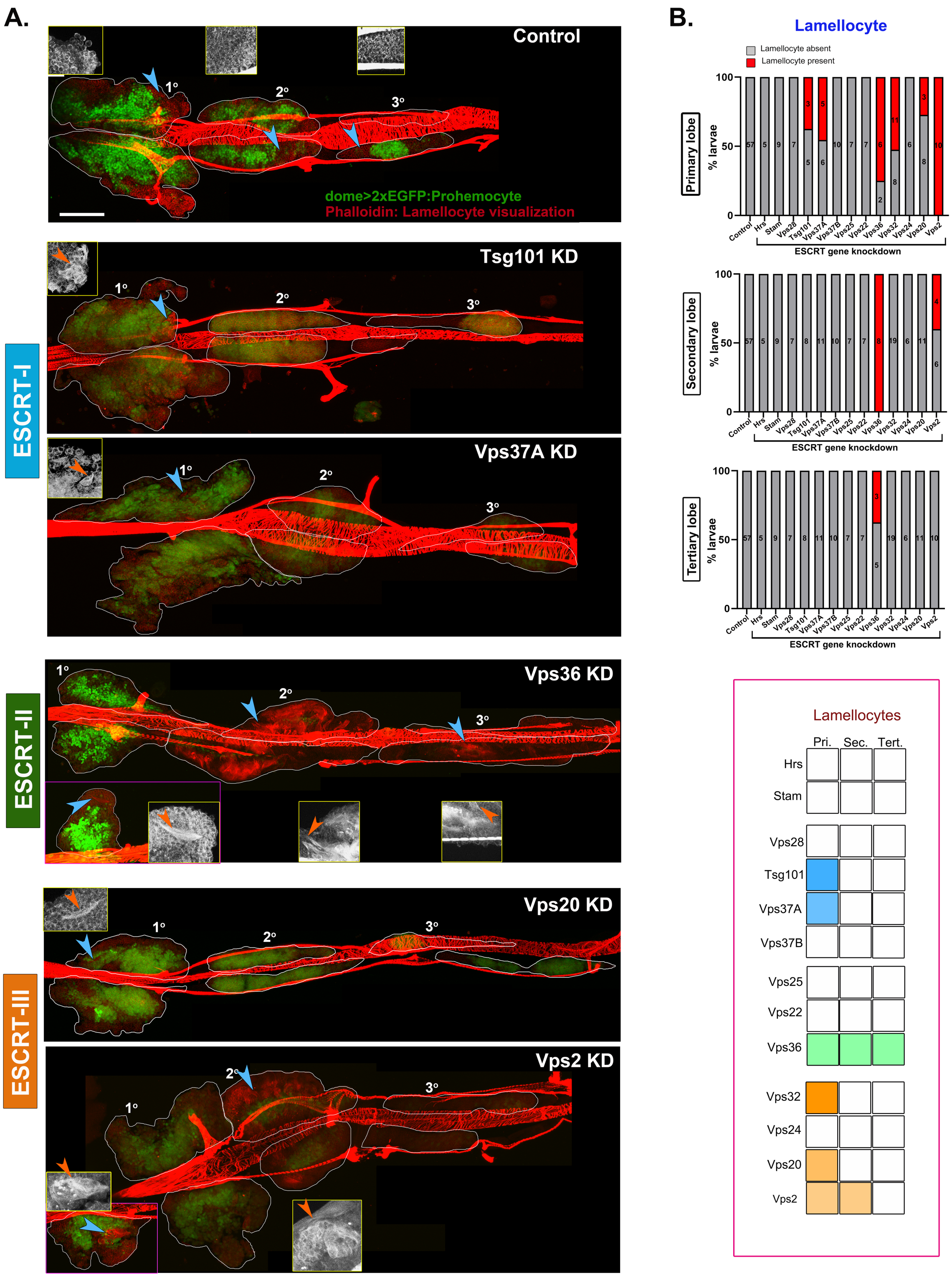

Supplementary Figure S9

Biology Open • Supplementary information

Fig. S9. ESCRT components regulate lamellocyte differentiation in the lymph gland.

(A) Whole-mount larval lymph gland showing Phalloidin staining (red) to visualise elongated morphology of lamellocytes upon progenitor-specific knockdown of 5 ESCRT components (Tsg101, Vps37A, Vps36, Vps20, Vps2). Blue arrowheads mark the region from primary, secondary or tertiary lobes, magnified in the insets. The inset panel shows enlarged view of Phalloidin staining with lamellocytes marked by orange arrowhead. Scale bar: 100 µm. **(B)** Bar diagram shows quantification of the percentage of lymph glands showing lamellocyte differentiation in primary, secondary and tertiary lobes upon knockdown of all 13 core ESCRT components, without any immune challenge. Values in the columns indicate the number of larvae analysed for presence or absence of lamellocytes. Summary chart indicating presence (colored box) or absence (white box) of lamellocytes differentiation in the primary, secondary and tertiary lobes upon depletion of the respective ESCRT component (left).

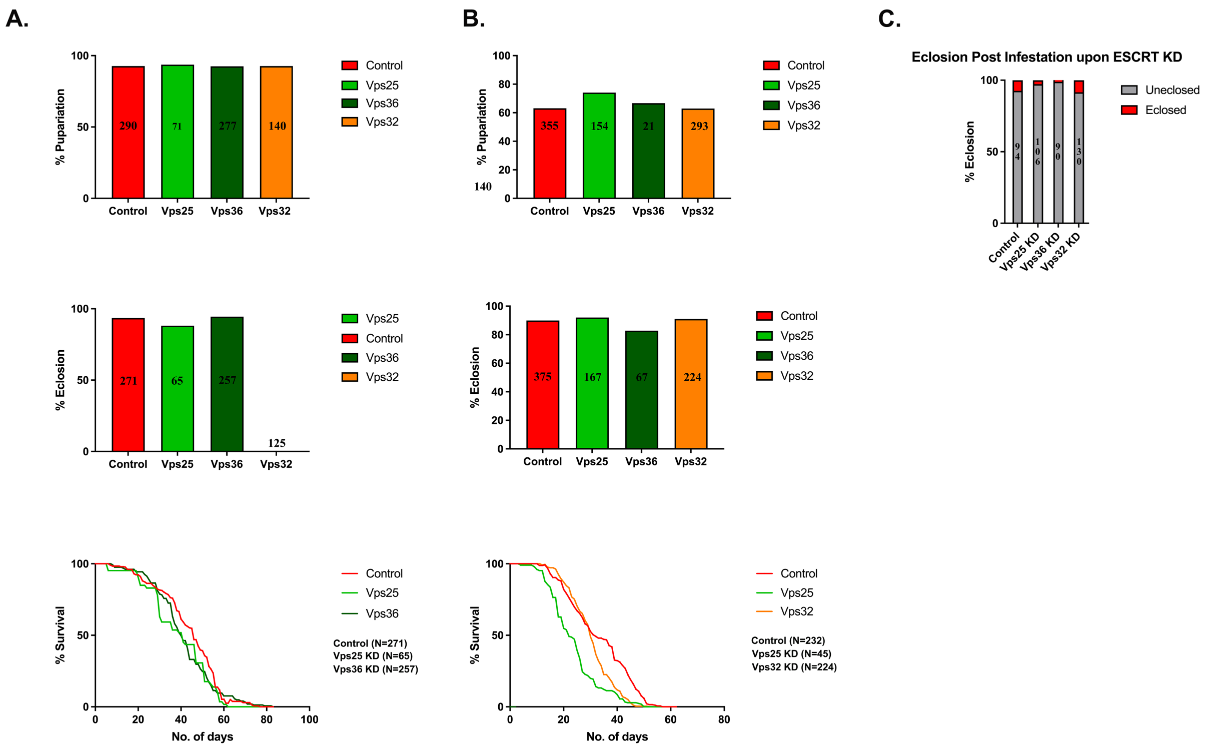

Supplementary Figure S10

Fig. S10. ESCRT depletion in hematopoietic compartments does not affect development and survival.

Developmental analysis of *Drosophila* with Vps25KD, Vps32KD and Vps36KD under (A) dome>2xEGFP and (B) elavGal80;;domeMESO-GFP> drivers. Quantification in the bar graphs indicate percentage pupariation and eclosion for each genotype. The survival curves visualize percentage of live adult flies on each day. (C) Quantification visualizes the percentage survival of adult flies post wasp infestation, for flies with Vps25KD, Vps36KD and Vps32KD under elavGal80;;domeMESO-GFP> driver. N>90 for each genotype.

Table S1.

Available for download at
https://journals.biologists.com/bio/article-lookup/doi/10.1242/bio.060412#supplementary-data

Biology Open • Supplementary information
